# Liver-Directed Cyp2e1 RNA Interference Attenuates Hepatotoxicity Induced by Triptolide, a Bioactive Diterpenoid from *Tripterygium wilfordii* Hook. f.

**DOI:** 10.3390/ph19071087

**Published:** 2026-07-15

**Authors:** Zijin Zhang, Wenzhao Jiang, Ruoyao Sang, Zhen Ouyang, Qian Wu, Yuan Wei

**Affiliations:** 1 School of Pharmacy, Jiangsu University, Zhenjiang 212013, China; zhangzijin2025@163.com (Z.Z.); jwzhao2023@163.com (W.J.); 155189537665@163.com (R.S.); zhenouyang@ujs.edu.cn (Z.O.); 2 Department of Laboratory Medicine, Tongren Hospital, Shanghai Jiao Tong University School of Medicine, 1111 Xianxia Rd, Shanghai 200336, China; 3 Haihe Laboratory of Cell Ecosystem, 10 Yuexin Rd, Tianjin 300400, China

**Keywords:** *Tripterygium wilfordii* Hook. f., triptolide, hepatotoxicity, CYP2E1, RNA interference, oxidative stress, pharmacokinetics

## Abstract

**Objectives:** Triptolide (TP), a bioactive diterpenoid from *Tripterygium wilfordii* Hook. f., has pharmacological activity, but repeated exposure is limited by hepatotoxicity. CYP2E1 is a hepatic metabolic-redox enzyme linked to oxidative liver injury. This study evaluated whether liver-directed *Cyp2e1* RNA interference mitigates TP-induced subacute hepatotoxicity without evidence of a marked reduction in short-term systemic TP exposure. **Methods:** *Cyp2e1*-targeting siRNA was encapsulated in lipid nanoparticles (si-*Cyp2e1* LNPs) and validated for hepatic CYP2E1 protein knockdown in female C57BL/6J mice. Subacute liver injury was induced by oral TP at 800 μg/kg/day for 7 days. Prophylactic and concurrent si-*Cyp2e1* regimens were evaluated using serum transaminases, gross liver morphology, H&E histopathology with blinded semi-quantitative scoring, oxidative-stress indices, RNA-seq, RT-qPCR, Western blotting, and exploratory LC-MS/MS pharmacokinetic analysis. **Results:** si-*Cyp2e1* LNPs showed favorable nanoscale properties and robust hepatic CYP2E1 protein knockdown. Both regimens reduced ALT/AST elevations, improved gross liver appearance and histological injury scores, decreased hepatic ROS and malondialdehyde, and restored glutathione and superoxide dismutase. Transcriptomic and molecular analyses indicated TP-associated PI3K/AKT activation and reduced PI3K/AKT phosphorylation after si-*Cyp2e1* treatment. Exploratory pharmacokinetic profiling showed a lower early plasma TP peak, whereas AUC0–t and AUC0–∞ appeared broadly comparable within the 0–3 h observation window. **Conclusions:** Liver-directed *Cyp2e1* silencing provided proof-of-concept hepatoprotection against repeated TP exposure, accompanied by redox recovery and attenuated stress-associated PI3K/AKT activation. Because CYP2E1 enzymatic activity, TP-derived reactive metabolites, and pathway causality were not directly tested, these findings should be interpreted as pathway-associated evidence requiring further validation.

## 1. Introduction

*Tripterygium wilfordii* Hook. f. (Celastraceae), commonly known as Lei Gong Teng in traditional Chinese medicine, is an ethnomedicinal plant used for inflammatory and autoimmune disorders. Triptolide (TP), a highly oxygenated diterpenoid epoxide isolated from *T. wilfordii*, is regarded as one of the principal bioactive constituents responsible for its anti-inflammatory, immunosuppressive, and antitumor activities [1,2,3,4]. However, the same compound that supports the therapeutic potential of *T. wilfordii*-derived preparations also contributes to their narrow therapeutic window. Hepatotoxicity remains one of the major safety barriers to the clinical and translational use of TP-containing therapies, particularly under repeated administration conditions that more closely resemble treatment for chronic immune-mediated diseases than a single-dose acute challenge model [5,6,7,8,9]. Thus, the central problem is not merely to demonstrate that TP can injure the liver, but to develop a targeted strategy that mitigates hepatic toxicity while preserving the therapeutic relevance of TP exposure.

Current hepatoprotective approaches for TP toxicity remain limited. Conventional anti-inflammatory or antioxidant interventions, such as glucocorticoids, glycyrrhizic acid derivatives, or N-acetylcysteine, may reduce downstream injury responses but do not precisely address an upstream hepatic driver of TP-associated oxidative injury. Metabolism-based detoxification strategies also require caution because TP disposition is closely associated with hepatic drug-metabolizing enzymes; for example, CYP3A modulation can strongly affect TP pharmacokinetics, and enhanced clearance may reduce toxicity at the possible cost of lowering exposure-dependent efficacy [10,11,12]. Therefore, an ideal detoxification strategy for TP-derived therapy should protect the liver through a mechanism-based target while avoiding a major reduction in systemic TP exposure.

CYP2E1 provides a rational upstream target for this purpose. Although TP is largely metabolized through CYP3A-related pathways, CYP2E1 is positioned at the interface between xenobiotic metabolism and oxidative liver injury. Aberrant CYP2E1 activity promotes reactive oxygen species (ROS) generation, lipid peroxidation, mitochondrial stress, and inflammatory amplification [13,14,15]. Previous studies have implicated CYP2E1 in alcoholic liver injury, acetaminophen-induced liver injury, and other oxidative stress-driven hepatic diseases, and CYP2E1 inhibition or protein knockdown has shown protective potential in these settings. Importantly, TP has also been reported to induce CYP2E1 and promote oxidative stress and inflammatory signaling, suggesting that the CYP2E1–redox axis may contribute to TP hepatotoxicity [16,17]. However, whether selective hepatic *Cyp2e1* silencing can attenuate TP-induced liver injury under repeated exposure has not been clearly established.

RNA interference (RNAi) offers a precise strategy for examining this hypothesis because it enables sequence-specific suppression of toxicity-related genes [18]. Naked siRNA is unsuitable for systemic application because of rapid degradation, poor cellular uptake, and rapid clearance. Lipid nanoparticles (LNPs), by contrast, are clinically advanced non-viral RNA delivery systems with strong liver tropism and established translational precedent for hepatic RNA delivery [3,19,20,21,22,23]. These features make LNP-mediated delivery of *Cyp2e1*-targeting siRNA a feasible approach to selectively reduce hepatic CYP2E1 protein expression and examine whether this intervention is accompanied by reduced CYP2E1-linked redox stress and attenuation of TP-induced hepatotoxicity.

Accordingly, this study was designed to evaluate liver-directed *Cyp2e1* RNA interference as a mechanism-informed, proof-of-concept hepatoprotective strategy against TP-induced subacute liver injury. We first prepared and characterized si-*Cyp2e1* LNPs and confirmed hepatic CYP2E1 protein knockdown in vivo. We then established a repeated-exposure TP model and evaluated both prophylactic and concurrent intervention schedules. To explore pathway-associated mechanisms, we integrated biochemical injury assessment, histopathology, hepatic redox markers, transcriptomic profiling, RT-qPCR, and Western blot validation of downstream signaling. Finally, because CYP2E1 is linked to xenobiotic metabolism, we performed an exploratory LC-MS/MS-based pharmacokinetic analysis to examine whether *Cyp2e1* silencing markedly reduces short-term systemic TP exposure. This design addressed two key questions: whether hepatic *Cyp2e1* silencing protects against repeated TP-induced hepatotoxicity, and whether this protection can be achieved without evidence of a marked reduction in short-term TP exposure within the tested window.

## 2. Results

### 2.1. si-Cyp2e1 LNPs Exhibited Favorable Physicochemical Properties and Efficiently Suppressed Hepatic CYP2E1 Expression in Vivo

**Figure 1:**
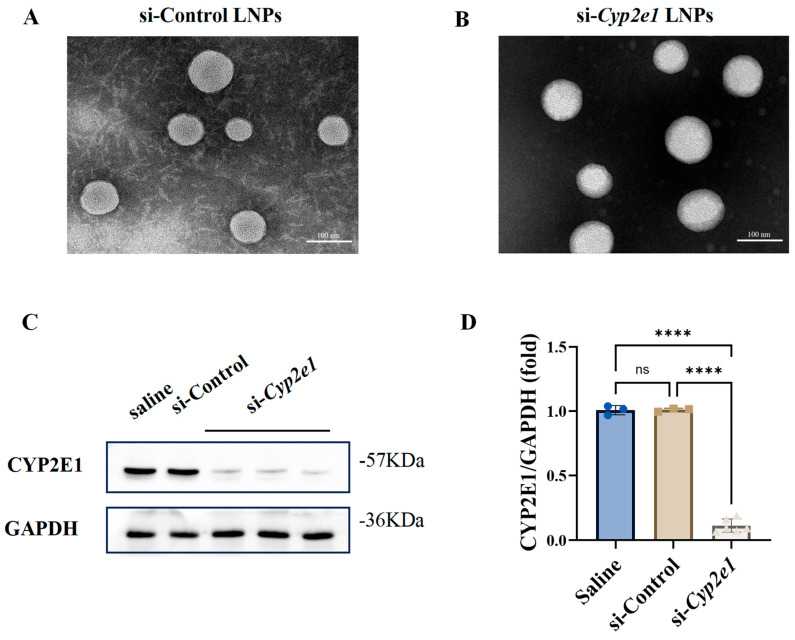
Characterization of si-*Cyp2e1* lipid nanoparticles and validation of hepatic CYP2E1 silencing in vivo. (**A**,**B**) Representative transmission electron microscopy (TEM) images of si-Control LNPs and si-*Cyp2e1* LNPs, respectively. Scale bar = 100 nm. (**C**) Representative Western blot showing hepatic CYP2E1 and GAPDH protein expression 24 h after a single intravenous injection of saline, si-Control LNPs, or si-*Cyp2e1* LNPs. (**D**) Densitometric quantification of CYP2E1 protein expression normalized to GAPDH. Data are expressed as mean ± SD. For panel D, *n* = 3 mice per group. Statistical significance is indicated as follows: ns, not significant; **** *p* < 0.0001.

We first evaluated whether the si-*Cyp2e1* LNP formulation possessed the physicochemical properties required for systemic siRNA delivery and hepatic target engagement. As shown in Table 1, si-*Cyp2e1* and si-Control LNPs displayed closely matched nanoscale characteristics, including comparable hydrodynamic diameter, polydispersity index, zeta potential, encapsulation efficiency, and loading capacity. si-*Cyp2e1* LNPs showed a mean particle size of 72.47 ± 1.15 nm, a PDI of 0.144 ± 0.008, a zeta potential of −0.45 ± 0.10 mV, an encapsulation efficiency of 82.89%, and a drug loading of 0.46 mg/mL. The corresponding values for si-Control LNPs were 71.26 ± 0.62 nm, 0.213 ± 0.006, −0.37 ± 0.10 mV, 75.31%, and 0.45 mg/mL, respectively. Transmission electron microscopy confirmed that both formulations were approximately spherical, well dispersed, and consistent with the nanoscale size distribution measured by dynamic light scattering (Figure 1A,B).

Next, we examined whether si-*Cyp2e1* LNPs effectively silenced hepatic CYP2E1 expression in vivo. Western blot analysis performed 24 h after a single intravenous injection showed that hepatic CYP2E1 protein abundance was markedly reduced in the si-*Cyp2e1* group, whereas saline and si-Control LNP administration did not suppress CYP2E1 expression (Figure 1C). Densitometric quantification further confirmed that si-*Cyp2e1* LNPs reduced hepatic CYP2E1 protein expression by approximately 90% compared with the control conditions (Figure 1D). These results demonstrated that the LNP formulation combined favorable physicochemical properties with robust hepatic target protein knockdown, thereby providing an appropriate RNAi tool for evaluating the relationship between *Cyp2e1* silencing and TP-induced subacute hepatotoxicity. This validation confirmed protein-level target engagement but did not directly assess CYP2E1 enzymatic activity, which was considered when interpreting the mechanistic data.

### 2.2. Hepatic Cyp2e1 Silencing Alleviated TP-Induced Subacute Liver Injury Under Both Prophylactic and Concurrent Treatment Paradigms

**Figure 2:**
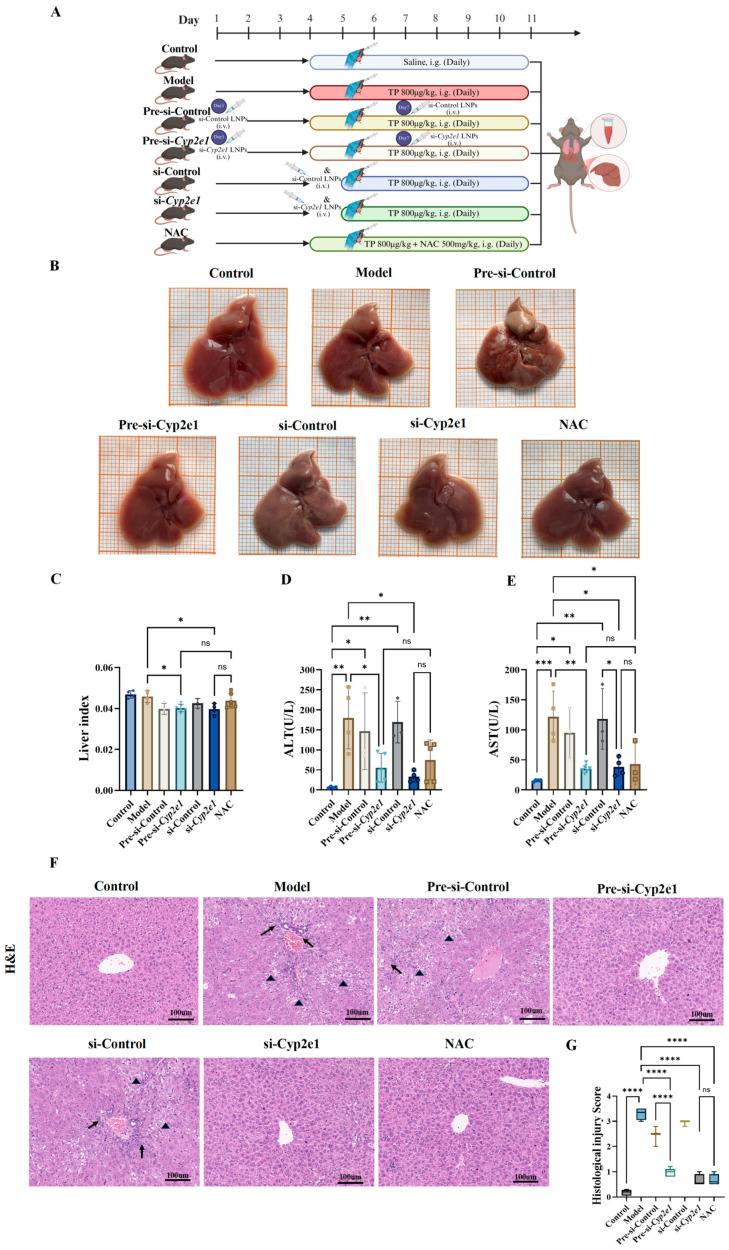
Liver-directed *Cyp2e1* silencing attenuates triptolide-induced subacute hepatotoxicity. (**A**) Schematic illustration of the experimental design for the Control, TP Model, Pre-si-Control, Pre-si-*Cyp2e1*, si-Control, si-*Cyp2e1*, and N-acetylcysteine (NAC) groups. (**B**) Representative gross liver appearance in each group. (**C**) Liver index. (**D**) Serum ALT levels. (**E**) Serum AST levels. (**F**) Representative H&E-stained liver sections. Arrows indicate hepatocellular degeneration or shrinkage, and arrowheads indicate inflammatory cell infiltration. Scale bar = 100 μm. (**G**) Blinded semi-quantitative histological injury score based on endpoint-available H&E-stained liver sections. Histological scoring was performed using a 0–4 scale according to hepatocellular degeneration or shrinkage, nuclear pyknosis or karyolysis, inflammatory cell infiltration, and disruption of hepatic architecture. For each evaluable animal, five randomly selected non-overlapping fields were assessed, and the average score was used as one biological replicate. For panels (**C**–**E**), data are expressed as mean ± SD from endpoint-available animals; because a small number of animals died before scheduled sample collection, sample sizes were not equal across all groups, and the plotted individual data points indicate the number of biological replicates used for each endpoint. For panel (**G**), box-and-whisker plots show semi-quantitative injury scores with individual animal values; the number of evaluable animals per group is indicated by the plotted data points. Panels (**C**–**E**) were analyzed by one-way ANOVA followed by Tukey’s multiple-comparison test. Panel (**G**) was analyzed using the Kruskal–Wallis test followed by Dunn’s multiple-comparison test. Statistical significance is indicated as follows: ns, not significant; * *p* < 0.05; ** *p* < 0.01; *** *p* < 0.001; **** *p* < 0.0001. Direct comparisons between NAC and the si-*Cyp2e1*-treated groups were included in the multiple-comparison analysis for liver index, ALT, AST, and histological injury score; no statistically significant differences were detected between NAC and either prophylactic or concurrent si-*Cyp2e1* treatment for these endpoints.

Having established an evaluable subacute injury window based on the preliminary dose-titration data (Table S7 and Supplementary Figure S1), we next examined whether hepatic *Cyp2e1* silencing could protect against TP-induced liver injury under two intervention schedules: prophylactic treatment before and during TP exposure and concurrent treatment initiated with TP administration (Figure 2A).

The gross liver morphology revealed a clear protective phenotype. Compared with the Control group, the livers from the Model, Pre-si-Control, and si-Control groups appeared darker, duller, and more morphologically abnormal. In contrast, both the Pre-si-*Cyp2e1* and si-*Cyp2e1* groups showed visibly improved liver appearance, approaching the gross phenotype observed in the Control group. NAC treatment produced similar macroscopic improvement and was used as an antioxidant hepatoprotective reference (Figure 2B).

The quantitative injury markers, analyzed using endpoint-available samples, further supported this protective effect. The liver index varied within a relatively narrow range across groups and therefore showed limited discriminatory power in this model (Figure 2C). In contrast, serum transaminases were sensitive indicators of hepatocellular injury. TP exposure markedly increased ALT and AST levels in the Model group, whereas both prophylactic and concurrent si-*Cyp2e1* LNP treatments significantly reduced these elevations (Figure 2D,E). Importantly, the corresponding si-Control groups remained broadly comparable to the Model group, indicating that the observed protection was sequence-dependent rather than attributable to LNP administration alone. NAC treatment also reduced ALT and AST levels, providing a positive reference for antioxidant hepatoprotection. Direct statistical comparisons were further performed between the NAC group and the si-*Cyp2e1*-treated groups. For liver index, ALT, and AST, no statistically significant differences were detected between NAC and either prophylactic or concurrent si-*Cyp2e1* treatment under the present experimental conditions. These findings indicate that NAC and si-*Cyp2e1* produced broadly comparable protection in the major biochemical injury endpoints, although the two interventions should be interpreted as mechanistically distinct.

The histopathological findings were consistent with the gross and biochemical results. Livers from the Model, Pre-si-Control, and si-Control groups showed overt tissue injury, including disrupted hepatic architecture, hepatocellular degeneration or shrinkage, and inflammatory cell infiltration. In contrast, both si-*Cyp2e1*-treated groups exhibited substantially preserved lobular structures and markedly attenuated pathological damage. NAC treatment produced comparable histological improvement (Figure 2F). To further support the representative H&E observations, blinded semi-quantitative scoring was performed using endpoint-available liver sections. The TP Model, Pre-si-Control, and si-Control groups showed higher histological injury scores than the Control group, confirming reproducible TP-induced hepatic injury. Both prophylactic and concurrent si-*Cyp2e1* treatment reduced the histological injury score compared with the TP Model and corresponding si-Control groups, and NAC treatment also reduced the histological injury score (Figure 2G). These semi-quantitative data were consistent with the representative H&E images and further supported the hepatoprotective effect of liver-directed *Cyp2e1* silencing in both preventive and concurrent treatment settings.

### 2.3. si-Cyp2e1 Restored Hepatic Redox Homeostasis in the TP-Induced Subacute Liver Injury Model

Because CYP2E1 is closely linked to oxidative stress and xenobiotic-induced redox injury, we next investigated whether the hepatoprotective effect of si-*Cyp2e1* was accompanied by restoration of hepatic redox homeostasis. TP exposure induced a coherent oxidative stress phenotype characterized by increased hepatic ROS and MDA levels, together with reduced GSH levels and SOD activity (Supplementary Figure S2A–D). These changes indicate that repeated TP administration not only causes biochemical and histological liver injury but also disrupts the balance between oxidative burden and endogenous antioxidant defense.

Both prophylactic and concurrent si-*Cyp2e1* interventions substantially reversed this redox imbalance. Compared with the Model group, the Pre-si-*Cyp2e1* and si-*Cyp2e1* groups showed lower ROS accumulation and reduced MDA levels, indicating attenuation of oxidative stress and lipid peroxidation. In parallel, GSH levels and SOD activity were restored toward control values, suggesting recovery of endogenous antioxidant capacity (Supplementary Figure S2A–D). In contrast, the corresponding si-Control groups retained most features of the TP-induced oxidative stress phenotype. NAC treatment produced a broadly similar rescue pattern consistent with its antioxidant activity.

These findings indicate that improvement of hepatic redox homeostasis was a prominent feature associated with *Cyp2e1* silencing. Thus, si-*Cyp2e1* treatment was accompanied by reduced overt liver injury, lower oxidative stress, limited lipid peroxidation, and improved antioxidant defense in the subacute TP injury model. These coordinated changes do not by themselves establish the complete causal sequence linking *Cyp2e1* suppression to hepatoprotection. To further explore signaling pathways associated with this protective phenotype, we performed transcriptomic profiling.

### 2.4. Transcriptomic Profiling Identified PI3K/AKT Signaling as a Candidate Stress-Associated Pathway Related to si-Cyp2e1-Mediated Hepatoprotection

To define the molecular programs associated with TP-induced liver injury and their attenuation by *Cyp2e1* silencing, we performed RNA sequencing using liver tissues from the Control, TP Model, and si-*Cyp2e1* LNP-treated groups. Differential expression analysis revealed extensive transcriptional remodeling after TP exposure and a substantial shift in the hepatic transcriptome following si-*Cyp2e1* treatment.

**Figure 3:**
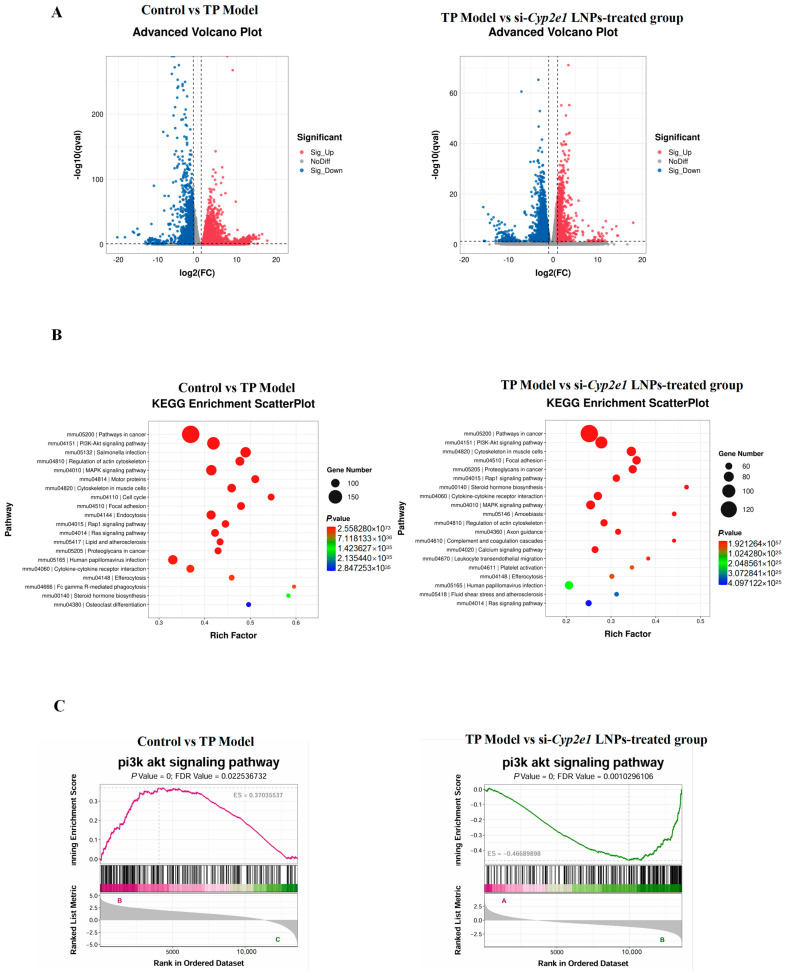
Transcriptomic profiling identified PI3K/AKT signaling as a candidate stress-associated pathway related to si-*Cyp2e1*-mediated hepatoprotection. (**A**) Volcano plots showing differentially expressed genes in the Control versus TP Model comparison and the TP Model versus si-*Cyp2e1* LNP-treated comparison. (**B**) Kyoto Encyclopedia of Genes and Genomes (KEGG) enrichment scatter plots for the two comparisons. (**C**) Gene set enrichment analysis (GSEA) plots showing enrichment of the PI3K/AKT signaling pathway in the TP Model group relative to the Control group and suppression of this pathway signature after si-*Cyp2e1* LNP treatment. Each comparison was performed using three biological replicates per group. RNA-seq workflow, clean-data quality, and reference-genome alignment metrics are provided in Tables S9 and S10. Additional enriched KEGG pathways and GO Biological Process terms beyond PI3K/AKT signaling, including adjusted *p* values/FDR values, are summarized in Tables S15A and S15B.

Compared with the control group, the TP Model group exhibited 6780 differentially expressed genes, including 5634 upregulated and 1146 downregulated transcripts. In contrast, a comparison between the TP Model and si-*Cyp2e1* LNP-treated groups identified 3551 differentially expressed genes, including 839 upregulated and 2712 downregulated genes. Volcano plots revealed broad TP-induced transcriptional disturbances, which were partially reversed after si-*Cyp2e1* treatment (Figure 3A).

In addition to PI3K/AKT signaling, enrichment analysis also suggested broader pathway-level disturbances involving inflammatory and immune-response programs, xenobiotic and lipid metabolism-related processes, extracellular matrix or cell adhesion-related remodeling, and oxidative stress- or lipid peroxidation-associated biological programs. Several of these pathway categories were shifted after si-*Cyp2e1* treatment, suggesting that the protective phenotype was associated with broader transcriptional recovery rather than modulation of a single downstream cascade. The selected enriched KEGG pathways and GO Biological Process terms for the Control versus TP Model and TP Model versus si-*Cyp2e1* comparisons are summarized in Tables S15A and S15B, with *p* values and adjusted *p* values/FDR values provided for each listed pathway or term.

Pathway enrichment analysis identified PI3K/AKT signaling as one of the pathways associated with both TP-induced liver injury and si-*Cyp2e1*-mediated transcriptional remodeling. KEGG enrichment analysis showed that PI3K/AKT signaling was enriched in both the Control versus TP Model comparison and the TP Model versus si-*Cyp2e1* LNP-treated comparison (Figure 3B). Consistently, GSEA indicated activation of the PI3K/AKT signaling signature in the TP Model group relative to the Control group, whereas this signature was suppressed after si-*Cyp2e1* treatment (Figure 3C).

Together, these transcriptomic findings indicate that PI3K/AKT signaling is a candidate stress-associated pathway involved in TP-induced subacute hepatotoxicity and attenuated by hepatic *Cyp2e1* silencing. Based on this pathway-level prediction, we performed targeted molecular validation.

### 2.5. si-Cyp2e1 Attenuated TP-Associated Activation of the PI3K/AKT Pathway

**Figure 4:**
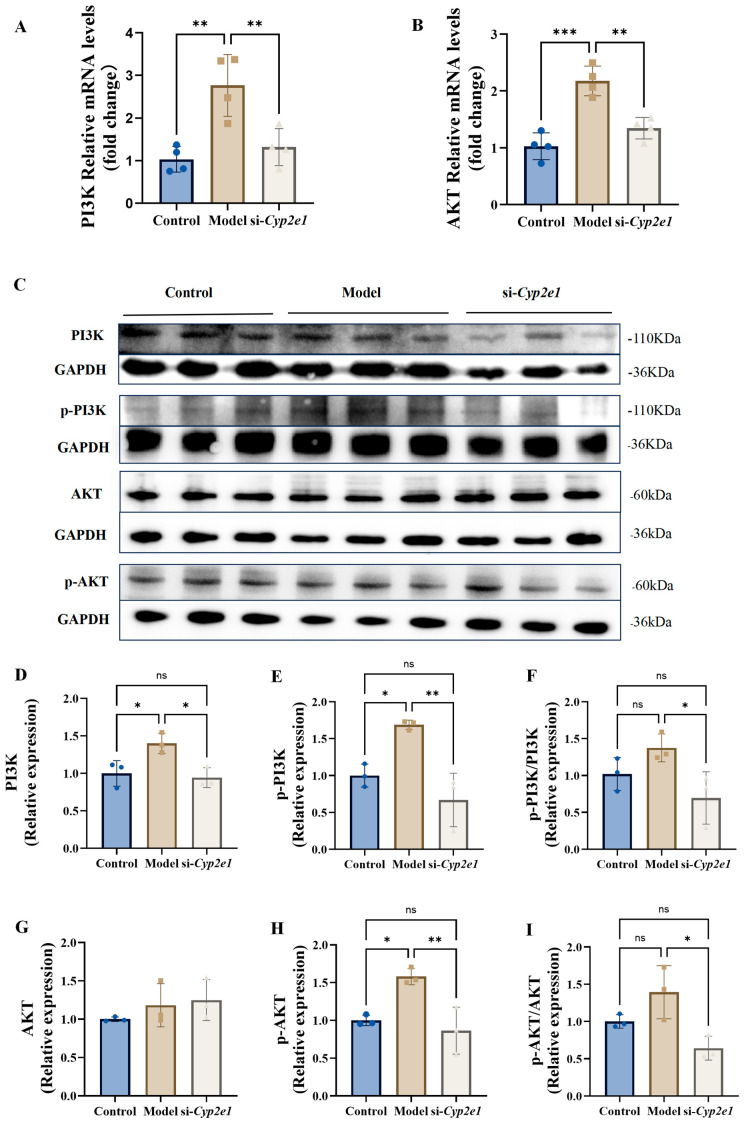
si-*Cyp2e1* attenuates triptolide-associated activation of the PI3K/AKT pathway. (**A**,**B**) RT-qPCR analysis of selected hepatic PI3K- and AKT-related mRNA signals. (**C**) Representative Western blots of PI3K, phosphorylated PI3K (p-PI3K), AKT, phosphorylated AKT (p-AKT), and GAPDH. (**D**) Relative PI3K protein expression. (**E**) Relative p-PI3K protein expression. (**F**) p-PI3K/PI3K ratio. (**G**) Relative AKT protein expression. (**H**) Relative p-AKT protein expression. (**I**) p-AKT/AKT ratio. mRNA expression was normalized to Gapdh, and protein expression was normalized to GAPDH. Data are expressed as mean ± SD. Statistical analysis was performed by one-way ANOVA followed by Tukey’s multiple-comparison test. Statistical significance is indicated as follows: ns, not significant; * *p* < 0.05; ** *p* < 0.01; *** *p* < 0.001.

Next, we validated whether the PI3K/AKT pathway, identified by transcriptomic analysis, was modulated at selected mRNA and protein levels. RT-qPCR analysis showed that selected PI3K- and AKT-related mRNA signals were higher in the TP Model group than in the Control group. si-*Cyp2e1* treatment reduced both signals, which was consistent with the RNA-seq-based pathway prediction (Figure 4A,B).

We further examined PI3K/AKT pathway activation at the protein level using Western blotting. TP exposure was accompanied by increased phosphorylation of PI3K and AKT, and si-*Cyp2e1* treatment reduced this phosphorylation response (Figure 4C). Densitometric analysis showed that total PI3K protein expression increased modestly in the Model group and declined after si-*Cyp2e1* treatment, whereas total AKT expression remained relatively stable across groups (Figure 4D,G). In contrast, p-PI3K and p-AKT levels were clearly elevated in the Model group and reduced after si-*Cyp2e1* treatment (Figure 4E,H).

To assess pathway activation more directly, phosphorylation ratios were calculated. Both the p-PI3K/PI3K and p-AKT/AKT ratios increased after TP exposure, indicating activation of the PI3K/AKT signaling axis. si-*Cyp2e1* treatment reduced both phosphorylation ratios (Figure 4F,I). These data suggest that the dominant effect of hepatic *Cyp2e1* silencing on this pathway occurred at the level of stress-associated phosphorylation and pathway activation rather than through broad suppression of total PI3K or AKT abundance.

Taken together, these results validated the transcriptomic prediction and indicated that si-*Cyp2e1* treatment was accompanied by attenuation of TP-associated PI3K/AKT pathway activation in a subacute liver injury model. However, because no pathway-specific PI3K/AKT inhibitor or activator was used, these data should be interpreted as pathway-associated evidence rather than proof that PI3K/AKT attenuation is required for the hepatoprotective effect of si-*Cyp2e1*. Given that CYP2E1 is also linked to xenobiotic metabolism, we next performed an exploratory pharmacokinetic analysis to examine whether hepatic *Cyp2e1* silencing markedly reduces short-term systemic TP exposure.

### 2.6. Exploratory LC–MS/MS-Based Pharmacokinetic Analysis Suggested a Lower Early TP Plasma Peak with Broadly Comparable Short-Term AUC After si-Cyp2e1 Treatment

**Figure 5:**
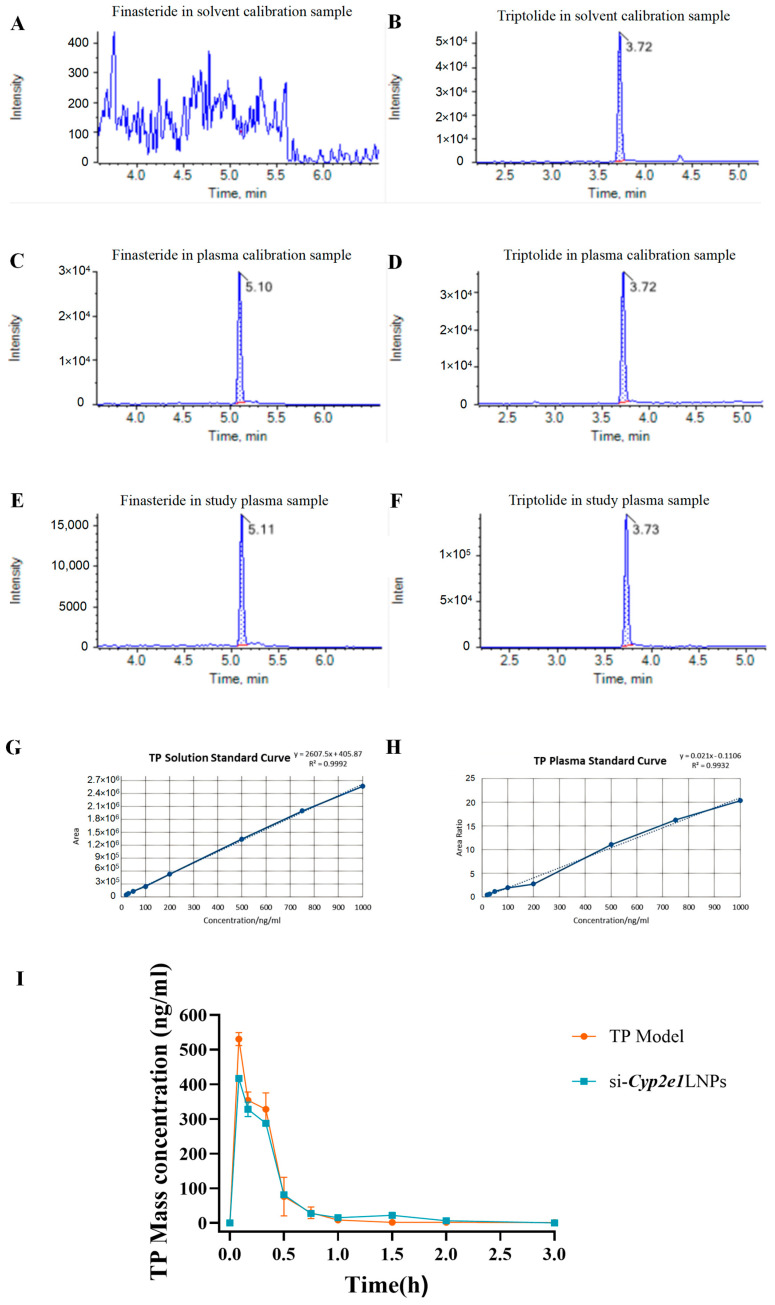
LC–MS/MS-based pharmacokinetic analysis of triptolide in TP Model and si-*Cyp2e1*-treated mice. (**A**) Representative chromatogram of finasteride, the internal standard, in a solvent calibration sample. (**B**) Representative chromatogram of triptolide (TP) in a solvent calibration sample. (**C**) Representative chromatogram of finasteride in a plasma calibration sample. (**D**) Representative chromatogram of TP in a plasma calibration sample. (**E**) Representative chromatogram of finasteride in a study plasma sample. (**F**) Representative chromatogram of TP in a study plasma sample. (**G**) TP calibration curve prepared in solvent. (**H**) TP calibration curve prepared in blank mouse plasma. (**I**) Mean plasma concentration–time profiles of TP after oral administration in the TP Model and si-*Cyp2e1* LNP-treated groups. Plasma concentration–time data are expressed as mean ± SD (n = 3 mice per group). Additional LC–MS/MS conditions and calibration information are provided in Table S14. Pharmacokinetic parameters were calculated by non-compartmental analysis and are summarized in Table 2.

Finally, we examined whether hepatic *Cyp2e1* silencing affected systemic TP exposure. Representative LC–MS/MS chromatograms showed clear detection of the internal standard finasteride and TP in the solvent calibration samples, plasma calibration samples, and study plasma samples (Figure 5A–F). The corresponding solution-phase and plasma calibration curves supported quantitative analysis of TP in mouse plasma (Figure 5G,H).

The mean plasma concentration–time profiles showed that TP was rapidly absorbed after oral administration in both groups, reaching peak plasma concentration at 0.08 h, followed by a rapid decline during the early post-dose phase (Figure 5I). Compared with the TP Model group, the si-*Cyp2e1* LNP-treated group showed a lower observed early plasma peak, together with slightly higher concentrations at later time points.

The non-compartmental pharmacokinetic analysis was interpreted descriptively. The observed Cmax was lower in the *si-Cyp2e1* LNP-treated group than in the TP Model group, with values of 417.31 ± 4.35 and 517.15 ± 14.06 ng/mL, respectively. Tmax remained 0.08 h in both groups. AUC0–t and AUC0–∞ values appeared broadly comparable between groups within the 0–3 h observation window, and apparent oral clearance (CLz/F) also appeared similar (Table 2). Because of the exploratory design, these findings should be interpreted cautiously and not as definitive evidence that systemic TP exposure or clearance was unchanged.

These exploratory pharmacokinetic data suggest that hepatic *Cyp2e1* silencing was associated with a lower observed early TP plasma peak, whereas AUC0–t, AUC0–∞, and apparent oral clearance appeared broadly comparable within the 0–3 h observation window. Because this pharmacokinetic module was descriptive, based on a limited sample size, and performed after a single TP dose, these findings should not be interpreted as definitive evidence that systemic TP exposure or clearance is unchanged. Rather, they suggest that *si-Cyp2e1* treatment did not cause a marked reduction in short-term systemic TP exposure under the present experimental conditions. The hepatoprotective phenotype was accompanied by CYP2E1 protein suppression, redox recovery, and reduced stress-associated PI3K/AKT pathway activation, but these coordinated changes remain associative. Collectively, the integrated formulation, efficacy, redox, transcriptomic, signaling, and exploratory pharmacokinetic data support hepatic *Cyp2e1* silencing as a pathway-informed, proof-of-concept RNAi strategy for mitigating TP-induced subacute hepatotoxicity.

## 3. Discussion

This study evaluated liver-directed *Cyp2e1* RNA interference as a mechanism-informed, proof-of-concept strategy for mitigating TP-induced subacute hepatotoxicity. Rather than reiterating each endpoint, the overall evidence supports three main interpretations: hepatic CYP2E1 protein suppression was associated with attenuation of TP-induced liver injury, this protection was accompanied by improved redox balance and reduced stress-associated PI3K/AKT phosphorylation, and the exploratory pharmacokinetic assessment did not show a marked reduction in short-term systemic TP exposure within the tested 0–3 h window. These findings support hepatic CYP2E1 as a functionally relevant contributor to TP-induced redox-associated liver injury, while not establishing a complete linear causal sequence from CYP2E1 suppression to PI3K/AKT modulation and hepatoprotection.

The present findings are consistent with, and extend, previous work implicating CYP2E1 in oxidative hepatotoxicity. CYP2E1 has been widely described as a metabolic-redox enzyme that can amplify ROS generation, lipid peroxidation, mitochondrial stress, and inflammatory signaling in alcoholic liver injury, acetaminophen-induced liver injury, and other oxidative stress-related hepatic disorders [13,14,15]. Previous TP-related studies also reported that TP exposure can induce CYP2E1 expression and promote oxidative and inflammatory liver injury, supporting the relevance of the CYP2E1–redox axis in TP hepatotoxicity [16,17]. Compared with those studies, the present work advances this concept by applying liver-directed *Cyp2e1* RNA interference in a repeated-exposure TP model rather than relying on nonspecific antioxidant intervention or pharmacological enzyme modulation. However, because CYP2E1 enzymatic activity and TP-derived reactive metabolites were not directly measured, our data should be interpreted as evidence supporting a CYP2E1-associated redox mechanism rather than definitive proof of reduced CYP2E1 catalytic bioactivation.

The repeated-exposure model is also important for interpreting the translational relevance of the study. TP-containing preparations are typically associated with repeated use in inflammatory or autoimmune settings, and TP toxicity is influenced by dose schedule, metabolic status, inflammatory background, and host factors [5,6,7,24]. By selecting an 800 μg/kg/day, 7-day regimen that produced reproducible biochemical and histological injury with acceptable tolerability, the present model provided a practical window for testing hepatoprotective intervention under conditions more relevant to continued TP exposure than acute overdose paradigms.

This study also relates to the broader development of liver-targeted RNAi therapeutics. LNPs and related hepatic RNA delivery systems have been widely explored because of their preferential liver accumulation and ability to deliver nucleic acids to hepatic cells [3,18,19,20,21,22,23]. In contrast to RNAi strategies designed to correct a disease-driving hepatic transcript, the present approach uses transient hepatic *Cyp2e1* silencing as a protective strategy against xenobiotic-induced injury. This distinction is important because CYP2E1 is involved not only in toxicant-associated redox stress but also in the metabolism of endogenous substrates and co-administered xenobiotics. Therefore, the therapeutic value of this approach will depend on achieving a balance between hepatoprotection, reversibility of CYP2E1 suppression, and preservation of necessary metabolic function.

The transcriptomic findings further support the view that TP-induced hepatotoxicity is multifactorial. In addition to PI3K/AKT signaling, GO and KEGG analyses indicated enrichment of inflammatory and immune-response pathways, xenobiotic and lipid metabolism, extracellular matrix or cell adhesion remodeling, and oxidative stress- or lipid peroxidation-associated programs. PI3K/AKT signaling was selected for molecular validation because it was enriched in both the TP-induced injury comparison and the si-*Cyp2e1* intervention comparison and was compatible with the CYP2E1–redox stress axis [25,26]. Nevertheless, the PI3K/AKT data should be interpreted as pathway-associated validation of a stress-response signature, not as proof that PI3K/AKT attenuation is required for hepatoprotection. Similarly, other enriched pathways, including inflammatory and lipid peroxidation-related programs, require dedicated validation before being assigned a causal role.

NAC and the exploratory pharmacokinetic module further help position the present strategy. NAC served as a downstream antioxidant reference, whereas si-*Cyp2e1* was designed to target an upstream CYP2E1-associated redox amplifier; therefore, comparable biochemical protection should not be interpreted as mechanistic equivalence. The pharmacokinetic data suggested a lower observed early TP plasma peak with broadly comparable short-term AUC values within the 0–3 h window, arguing against a marked reduction in short-term systemic exposure as the sole explanation for hepatoprotection. Because this analysis was descriptive, based on n = 3 mice per group, and limited to a single-dose short sampling window, it should not be interpreted as definitive evidence that TP disposition is unchanged under broader dosing conditions.

From a translational perspective, this strategy should be regarded as proof-of-concept rather than an immediately applicable clinical regimen. Although LNPs provide an efficient platform for hepatic RNA delivery, intravenous administration, repeated dosing, infusion-related reactions, complement activation, anti-PEG responses, and immunostimulatory effects of lipid or siRNA components remain important considerations, particularly for chronic autoimmune or inflammatory diseases requiring long-term treatment. Future studies should define the dosing interval, reversibility of CYP2E1 suppression, tissue biodistribution, carrier-specific hepatotoxicity, immunogenicity, repeated-dose safety, and possible interactions with CYP2E1-metabolized drugs before clinical translation can be considered.

This study had several limitations. First, hepatic CYP2E1 protein suppression was robust, but CYP2E1 enzymatic activity and TP-derived reactive metabolites were not directly assessed; therefore, the current data demonstrate target protein knockdown rather than direct functional inhibition of CYP2E1 catalytic activity. Second, the observed changes in CYP2E1 protein expression, oxidative stress markers, PI3K/AKT phosphorylation, and liver injury endpoints remain largely associative. Pathway-specific gain- or loss-of-function experiments are needed to determine whether PI3K/AKT modulation is causally required. Third, the pharmacokinetic analysis was exploratory and descriptive, with a limited sample size, a single-dose design, and a short 0–3 h observation window; repeated-dose pharmacokinetics, extended sampling, hepatic TP concentrations, and TP metabolite profiling were not assessed. Fourth, although si-Control LNP groups did not show evidence of additional aggravation of TP-induced liver injury in the present efficacy study, carrier-specific safety in the absence of TP was not comprehensively evaluated. Finally, only female mice were used to maintain consistency with the dose-optimization experiments and to reduce aggression- or stress-related variability during repeated treatment. Whether the protective effect extends to male animals, longer TP exposure schedules, or disease-background models remains to be determined.

## 4. Materials and Methods

### 4.1. Animals

Healthy female C57BL/6J mice (6–8 weeks old, 18–22 g) were obtained from the Experimental Animal Center of Jiangsu University (Jiangsu, China). Only female mice were used in this proof-of-concept study to maintain consistency with the preliminary dose-optimization experiments and to reduce aggression-related injury and stress-related variability during repeated treatment. Animals were maintained under specific pathogen-free conditions at 22 ± 2 °C and 55 ± 5% relative humidity on a 12 h light/12 h dark cycle, with free access to standard chow and water. All animal experiments were approved by the Institutional Animal Care and Use Committee of Jiangsu University (approval number: UJS-IACUC-2025021902; approval date: 19 February 2025) and performed in accordance with institutional guidelines for the care and use of laboratory animals under Laboratory Animal Use License No. SYXK (Su) 2023-0081.

### 4.2. Chemicals, Reagents, and Compound Information

The full botanical name *Tripterygium wilfordii* Hook. f. (Celastraceae) was verified against the World Flora Online database for October 2024. Commercially obtained triptolide (TP), a representative bioactive and toxicity-associated diterpenoid epoxide derived from *T. wilfordii*, was used in this study. TP (CAS No. 38748-32-2; molecular formula C_20_H_24_O_6_; molecular weight 360.41; purity ≥98% by HPLC; catalog No. A0104) was purchased from Must Bio-Technology Co., Ltd. (Chengdu, China), and its purity was confirmed using a supplier-provided certificate of analysis. N-acetylcysteine and finasteride were used as the positive hepatoprotective reference and LC–MS/MS internal standard, respectively; detailed compound information is provided in Table S1. Additional reagents, assay kits, instruments, and consumables are summarized in Tables S2 and S3.

A TP stock solution was prepared by dissolving TP (12.5 mg) in dimethyl sulfoxide (DMSO) 5 mL to a concentration of 2.5 mg/mL. The solution was vortexed until complete dissolution, aliquoted at 100 μL per tube, and stored at −80 °C protected from light. Immediately prior to administration, the TP stock solution was freshly diluted to the target dose with sterile normal saline. The final vehicle composition was consistent across the treatment groups.

### 4.3. Preparation and Characterization of si-Cyp2e1 LNPs

si-*Cyp2e1*-loaded lipid nanoparticles (si-*Cyp2e1* LNPs) were prepared by microfluidic mixing according to established principles for ionizable lipid nanoparticle assembly and RNA encapsulation [3,19,20,21,22,23]. Briefly, DLin-MC3-DMA, cholesterol (AVT, Shanghai, China), DSPC (Macklin, Shanghai, China), and 14:0 PEG2000 PE (Sigma-Aldrich, St. Louis, MO, USA) were dissolved in anhydrous ethanol at a molar ratio of 50:38.5:10:1.5 to form the lipid phase. The aqueous phase consisted of siRNA dissolved in 10 mM sodium acetate buffer. The two phases were mixed on a microfluidic chip (Pengzan Biotechnology, Shanghai, China) at a volume ratio of 1:3 using a dual-channel syringe pump to allow spontaneous nanoparticle self-assembly. The resulting suspension was immediately transferred to a dialysis bag (MWCO 20 kDa) and dialyzed against RNase-free water for 2 h in the dark to remove residual ethanol. Chemically modified siRNA duplexes containing 2′-O-methyl modifications and 3′-dTsdT overhangs with phosphorothioate linkages, as specified in Table S4, were synthesized by GenScript Biotech (Jiangsu, China). Additional formulation, preparation, and characterization parameters are listed in Table S5.

After purification, nanoparticles were diluted in Dulbecco’s phosphate-buffered saline for physicochemical characterization. The hydrodynamic diameter, polydispersity index (PDI), and zeta potential were measured using a NanoBook 90Plus PALS instrument (Brookhaven Instruments, Holtsville, NY, USA). The morphology was examined using transmission electron microscopy (TEM; HT7800, Hitachi, Tokyo, Japan) after negative staining on carbon-coated copper grids.

siRNA encapsulation efficiency (EE%) was determined using the Quant-iT™ RiboGreen^®^ RNA Assay Kit (Thermo Fisher Scientific, Waltham, MA, USA). Total and free siRNAs were quantified in the presence or absence of 2% Triton X-100 (Biosharp, Anhui, China), and EE% was calculated from the difference between the two fluorescence measurements.

### 4.4. In Vivo Silencing Efficiency of si-Cyp2e1 LNPs

To evaluate hepatic silencing efficiency in vivo, healthy female C57BL/6J mice were randomly assigned to saline, si-Control LNP, and si-*Cyp2e1* LNP groups (n = 3 per group). The saline group received DPBS via tail vein injection, whereas the si-Control and si-*Cyp2e1* groups received the corresponding LNP formulations at 0.5 mg/kg siRNA equivalent. At 24 h after administration, the mice were anesthetized and euthanized, and liver tissues were rapidly harvested for protein analysis.

Approximately 50 mg of liver tissue was homogenized in lysis buffer containing radioimmunoprecipitation assay (RIPA) buffer, phenylmethylsulfonyl fluoride (PMSF), and protease inhibitor. After incubation on ice for 20 min, the homogenates were centrifuged at 13,000 rpm for 15 min at 4 °C, and the supernatants were collected for protein quantification. Equal amounts of protein were mixed with 5× sodium dodecyl sulfate-polyacrylamide gel electrophoresis (SDS-PAGE) loading buffer, denatured by heating, separated by SDS-PAGE, transferred onto polyvinylidene fluoride (PVDF) membranes, and blocked with 5% skim milk. Membranes were then incubated sequentially with the indicated primary and HRP-conjugated secondary antibodies. The in vivo silencing validation design is summarized in Table S6. Protein bands were visualized by enhanced chemiluminescence and quantified using ImageJ software (version 1.54t; National Institutes of Health, Bethesda, MD, USA).

### 4.5. Establishment of the Subacute Liver Injury Model and Treatment Protocols

Preliminary dose-optimization experiments were conducted to identify a repeated TP exposure regimen capable of producing a reproducible and evaluable subacute liver injury phenotype while preserving sufficient tolerability for intervention studies. Dose selection was based on combined biochemical, gross morphological, histopathological, and tolerability criteria, including serum transaminase elevation, visible liver appearance, H&E-confirmed liver injury, and mortality or severe toxicity. In the preliminary screening, lower-dose regimens produced minimal or insufficiently stable liver injury, whereas excessive TP exposure caused unacceptable toxicity and was not suitable for therapeutic evaluation. Based on these pilot experiments, TP at 800 μg/kg/day for 7 consecutive days produced reproducible biochemical and histological liver injury without excessive mortality and was therefore selected for all subsequent efficacy and mechanistic analyses.

Mice were randomly assigned to seven groups (initially n = 6 per group): Control, TP Model, Pre-si-Control, Pre-si-*Cyp2e1*, si-Control, si-*Cyp2e1*, and NAC (N-acetylcysteine). Subacute liver injury was induced by intragastric administration of TP at 800 μg/kg/day from day 4 to day 10, whereas mice in the Control group received an equal volume of normal saline. Because a small number of animals died before the scheduled endpoint during the subacute study, endpoint analyses were performed using samples obtained from endpoint-available animals, and no artificial values were assigned for animals without evaluable samples. The dose-screening information supporting model selection is summarized in Table S7 and Supplementary Figure S1. Detailed grouping and the treatment schedule are summarized in Table S8.

For prophylactic intervention, mice received intravenous injections of si-Control LNPs or si-*Cyp2e1* LNPs (0.5 mg/kg siRNA equivalent) on days 1 and 7, before and during TP exposure. For concurrent intervention, mice received a single intravenous injection of the corresponding LNP formulation at the same dose on day 4, coinciding with the initiation of TP administration. Mice in the NAC group received NAC via intragastric administration at 500 mg/kg/day from days 4 to 10 together with TP treatment. NAC was included as a pharmacological reference for antioxidant hepatoprotection rather than as a mechanistically equivalent comparator.

Blood was collected from the retro-orbital plexus under deep anesthesia 24 h after the last TP administration, after which the animals were euthanized and liver tissues were harvested. Liver samples were snap-frozen in liquid nitrogen for biochemical and molecular analyses or fixed in 4% paraformaldehyde for histopathological examination.

### 4.6. Serum Biochemical Analysis

Whole blood samples were allowed to stand at room temperature for 1 h and then centrifuged at 3500 rpm for 15 min to obtain the serum. Serum alanine aminotransferase (ALT) and aspartate aminotransferase (AST) levels were measured using commercial assay kits according to the manufacturer’s instructions and were used as quantitative indicators of hepatocellular injury.

### 4.7. Histopathological Examination

A representative liver lobe from each mouse was fixed in neutral fixative, embedded in paraffin, sectioned, and stained with hematoxylin and eosin (H&E). Histological images were acquired using a digital slide scanner. Liver injury was evaluated based on the hepatic lobular architecture, hepatocellular swelling or degeneration, nuclear pyknosis or karyolysis, and inflammatory cell infiltration.

In addition to representative image assessment, semi-quantitative histological injury scoring was performed in a blinded manner using endpoint-available H&E-stained liver sections. For each evaluable animal, five randomly selected non-overlapping fields were assessed, and the average score was used as one biological replicate. Liver injury was scored on a 0–4 scale according to hepatocellular degeneration or shrinkage, nuclear pyknosis or karyolysis, inflammatory cell infiltration, and disruption of hepatic architecture: 0, no obvious abnormality; 1, minimal injury; 2, mild injury; 3, moderate injury; and 4, severe injury. Animals without evaluable H&E sections because of death before scheduled tissue collection or inadequate tissue quality were not assigned artificial histological scores.

### 4.8. Assessment of Hepatic Oxidative Stress

Approximately 50 mg of liver tissue was homogenized in lysis buffer. Hepatic levels of ROS, malondialdehyde (MDA), reduced glutathione (GSH), and superoxide dismutase (SOD) were measured using the corresponding commercial assay kits, according to the manufacturer’s instructions. ROS and MDA were used to reflect oxidative stress and lipid peroxidation, respectively, whereas GSH and SOD were used to evaluate endogenous antioxidant capacity.

### 4.9. RNA Sequencing and Bioinformatic Analysis

To investigate the molecular basis of si-*Cyp2e1*-mediated protection against TP-induced subacute liver injury, liver tissues from the Control, TP Model, and si-*Cyp2e1* LNP-treated groups were subjected to transcriptomic profiling with three biological replicates per group. Approximately 100 mg fresh liver tissue from each mouse was snap-frozen in liquid nitrogen and transported on dry ice before sequencing. Total RNA was extracted using TRIzol reagent, and RNA concentration, purity, and integrity were assessed before library construction. Qualified samples were used for mRNA enrichment, cDNA library preparation, and high-throughput sequencing on an Illumina NovaSeq 6000 platform in the PE150 mode.

Raw sequencing reads were filtered to obtain clean data and aligned to the mouse reference genome. Gene expression was quantified using HTSeq (version 2.1.2) and StringTie (version 3.0.1). Differential-expression analysis was performed using the Limma method. Genes with *p* < 0.05 and fold change > 1.5 were retained as exploratory differentially expressed genes [27]. Multiple-testing correction was performed using the Benjamini–Hochberg procedure, and adjusted *p* values/FDR values were reported for the selected GO and KEGG enrichment results. Functional enrichment analyses, including Gene Ontology (GO), Kyoto Encyclopedia of Genes and Genomes (KEGG), and gene set enrichment analysis (GSEA), were used to identify pathways associated with TP-induced liver injury and their modulation by si-*Cyp2e1* treatment [28,29]. These transcriptomic analyses were used for pathway-level hypothesis generation and downstream molecular validation. The RNA-seq design, sample-level sequencing quality, clean-data metrics, reference-genome alignment metrics, bioinformatic workflow, and raw data quality are summarized in Tables S9 and S10.

### 4.10. RT-qPCR Analysis

Total RNA was extracted from approximately 50 mg of liver tissue using TRIzol reagent. After quality assessment, cDNA was synthesized according to the manufacturer’s instructions. Quantitative real-time PCR was performed using the SYBR Green qPCR Mix with GAPDH as the internal reference gene. Each 20 μL reaction contained 10.0 μL of 2× SYBR Green qPCR Mix, 0.4 μL each of forward and reverse primers, 1.0 μL of cDNA template, and 8.2 μL of nuclease-free water. The complete RT-qPCR reaction system is summarized in Table S12. Cycling conditions were 95 °C for 120 s, followed by 45 cycles of 95 °C for 30 s, 60 °C for 30 s, and 72 °C for 30 s. All samples were analyzed in triplicate, and relative mRNA expression was calculated using the 2^−ΔΔCt method with attention to contemporary qPCR reporting standards [30]. Based on the transcriptomic results, selected PI3K/AKT pathway-related and inflammation-related transcripts were analyzed. The primer sequences used for RT-qPCR validation were synthesized by Sangon Biotech (Shanghai, China) and are listed in Table S11.

### 4.11. Western Blot Analysis

Approximately 50 mg of liver tissue was homogenized in lysis buffer containing RIPA buffer, PMSF, and protease inhibitors. After incubation on ice for 20 min, the homogenates were centrifuged at 13,000 rpm for 15 min at 4 °C, and the supernatants were collected for protein quantification. Equal amounts of protein were mixed with 5× SDS-PAGE loading buffer, denatured by heating, separated by SDS-PAGE, transferred onto PVDF membranes, and blocked with 5% skim milk. Membranes were then incubated sequentially with the indicated primary and HRP-conjugated secondary antibodies. Protein bands were visualized by enhanced chemiluminescence and quantified using ImageJ. Information on the primary antibodies used for Western blotting is provided in Table S13.

For in vivo silencing validation, hepatic CYP2E1 protein expression was determined. For mechanistic validation, the protein levels of PI3K, phosphorylated PI3K (p-PI3K), AKT, and phosphorylated AKT (p-AKT) were analyzed to assess PI3K/AKT pathway activation. Phosphorylated and total protein levels were normalized to those of GAPDH, and the pathway activation ratios were calculated as p-PI3K/PI3K and p-AKT/AKT.

### 4.12. Exploratory Pharmacokinetic Study of TP

As an exploratory short-term assessment of whether hepatic *Cyp2e1* silencing markedly reduces short-term systemic TP exposure, a comparative LC–MS/MS-based pharmacokinetic study was performed in the TP Model group and si-*Cyp2e1* LNP-treated group, drawing on established LC–MS/MS and pharmacokinetic workflows for TP quantification [26,27,28]. Female C57BL/6J mice were fasted for 12 h before dosing with free access to water. Mice in the si-*Cyp2e1* group received the siRNA formulation via tail vein injection 72 h before TP administration. TP was then administered by oral gavage at 800 μg/kg in both groups. Blood samples were collected in EDTA-containing tubes at 5, 10, 20, 30, and 45 min, and at 1, 1.5, 2, and 3 h after dosing. Plasma was separated by centrifugation at 4000 rpm for 10 min and stored at −80 °C until analysis.

For TP quantification, 100 μL of plasma was mixed with 10 μL of finasteride internal standard solution (10 ng/mL) and extracted with 990 μL of ethyl acetate. After vortexing and centrifugation, the organic phase was collected, evaporated to dryness at 45 °C, and reconstituted in 60 μL of 80% methanol. The reconstituted samples were centrifuged and the resulting supernatants were subjected to LC–MS/MS analysis. Calibration standards were prepared by spiking blank mouse plasma with TP and processing in parallel with the study samples.

Chromatographic separation was performed on a ZORBAX SB-C18 column (3.0 × 100 mm, 3.5 μm) at 40 °C with a flow rate of 0.3 mL/min, an autosampler temperature of 4 °C, and an injection volume of 5 μL. The mobile phase consisted of methanol (A) and 0.05 mmol/L ammonium acetate (B) with the following gradient: 0–3.4 min, 50–95% A; 3.4–3.9 min, 95% A; 3.9–4.5 min, 95–50% A; and 4.5–7.0 min, 50% A. Mass spectrometric detection was carried out in positive electrospray ionization mode using multiple reaction monitoring and finasteride as the internal standard [10,11,12]. Plasma concentration–time profiles were constructed from three biologically independent profiles per group (n = 3 per group), and pharmacokinetic parameters were calculated by non-compartmental analysis using DAS 2.0. The primary descriptive parameters were observed Cmax and Tmax, AUC0–t, AUC0–∞, and apparent oral clearance (CLz/F) within the 0–3 h sampling window. Additional LC–MS/MS conditions and calibration information are listed in Table S14.

### 4.13. Statistical Analysis

All non-pharmacokinetic data, except semi-quantitative histological injury scores, are presented as mean ± standard deviation (SD). Statistical analyses were performed using GraphPad Prism 9.5. Differences among multiple groups were evaluated using one-way analysis of variance (ANOVA) followed by Tukey’s multiple comparison test, and statistical significance was set at *p* < 0.05.

Endpoint analyses were conducted using available biological samples collected at the scheduled endpoint; missing values caused by death before tissue collection or inadequate sample quality were not imputed. Semi-quantitative histological injury scores were analyzed using the Kruskal–Wallis test followed by Dunn’s multiple-comparison test. For histological scoring, each animal represented one biological replicate, and the actual number of endpoint-available evaluable animals per group is indicated by the plotted individual data points in the figure.

For the pharmacokinetic module, plasma TP concentrations and derived pharmacokinetic parameters were summarized descriptively as mean ± SD (n = 3 per group). Pharmacokinetic parameters were calculated by non-compartmental analysis using DAS 2.0. Given the exploratory design, limited sample size, and short 0–3 h sampling window, these results were interpreted descriptively rather than as formal inferential comparisons of non-compartmental parameters or definitive evidence of unchanged TP disposition.

## 5. Conclusions

In conclusion, liver-directed si-*Cyp2e1* LNPs effectively suppressed hepatic CYP2E1 protein expression and mitigated TP-induced subacute hepatotoxicity in mice. This protective phenotype was associated with reduced serum transaminase levels, improved liver histology, restoration of hepatic redox homeostasis, and attenuation of stress-associated PI3K/AKT pathway activation. Exploratory pharmacokinetic analysis further suggested that *Cyp2e1* silencing reduced the observed early TP plasma peak, whereas short-term AUC appeared broadly comparable within the 0–3 h measurement window. Because CYP2E1 enzymatic activity and TP-derived reactive metabolites were not directly measured, and pathway-specific PI3K/AKT intervention was not performed, the mechanistic findings should be interpreted as pathway-associated evidence rather than proof of a complete causal sequence or direct CYP2E1 functional inhibition. These findings support hepatic CYP2E1 as a functionally relevant contributor in TP-induced liver injury and support LNP-mediated *Cyp2e1* RNAi as a proof-of-concept hepatoprotective strategy, although carrier-specific hepatotoxicity, repeated-dose safety, immunogenicity, biodistribution, and clinical feasibility require further evaluation before translational application.

## Figures and Tables

**Table 1:** Physicochemical characterization of siRNA-loaded LNPs.

Formulation	Particle Size (nm)	PDI	Zeta Potential (mV)	Encapsulation Efficiency (%)	Drug Loading (mg/mL)
si-*Cyp2e1* LNPs	72.47 ± 1.15	0.144 ± 0.008	−0.45 ± 0.10	82.89	0.46
si-Control LNPs	71.26 ± 0.62	0.213 ± 0.006	−0.37 ± 0.10	75.31	0.45

**Table 2:** Exploratory descriptive short-term pharmacokinetic parameters of TP after oral administration in TP Model and *si-Cyp2e1* LNP-treated mice.

Parameter	Unit	TP Model	si-*Cyp2e1* LNP-Treated
Observed Cmax	ng/mL	517.15 ± 14.06	417.31 ± 4.35
Observed Tmax	h	0.08	0.08
AUC0–t	ng·h/mL	168.45 ± 17.70	166.25 ± 2.15
AUC0–∞	ng·h/mL	168.54 ± 17.69	171.46 ± 2.05
CLz/F	×10^3^ mL/h/kg	4.78 ± 0.51	4.67 ± 0.06

Data are presented as mean ± SD (n = 3 per group). Pharmacokinetic parameters were calculated by non-compartmental analysis using DAS 2.0. Because of the exploratory design, limited sample size, and short 0–3 h sampling window, these parameters were summarized descriptively and were not used to make definitive inferential claims regarding unchanged systemic exposure or clearance.

## Data Availability

The original contributions presented in the study are included in the article and Supplementary Material, further inquiries can be directed to the corresponding authors. The raw RNA-seq data have been deposited in the NCBI under BioProject number PRJNA1475773.

## Supplementary Materials

The following supporting information can be downloaded at: https://www.mdpi.com/article/10.3390/ph19071087/s1; Figure S1: Dose-titration data supporting the selection of 800 μg/kg/day TP for 7 days as the subacute liver injury regimen. Female C57BL/6J mice were orally administered TP at 600 or 800 μg/kg/day for 7 consecutive days, and samples were collected 24 h after the last dose; Figure S2: si-Cyp2e1 restored hepatic redox homeostasis in the TP-induced subacute liver injury model; Table S1: Compound information for the key chemicals used in this study; Table S2: Additional reagents and assay kits used in this study; Table S3: Major instruments and consumables used in this study; Table S4: siRNA sequences used for Cyp2e1 knockdown and negative control; Table S5: Additional formulation, preparation, and characterization parameters of siRNA-loaded lipid nanoparticles; Table S6: In vivo hepatic silencing validation design.; Table S7: Preliminary dose-titration experiments for establishing the repeated-exposure TP-induced subacute liver injury model; Table S8: Experimental grouping and treatment schedule for the subacute hepatoprotection study; Table S9: RNA sequencing design, quality control, and bioinformatic workflow; Table S10: RNA-seq sample-level data quality and alignment metrics; Table S11: Primer sequences used for RT-qPCR validation; Table S12: RT-qPCR reaction system; Table S13: Primary antibodies used for western blot analysis; Table S14: LC-MS/MS sample collection, calibration, and quantitative conditions; Table S15A. Selected enriched KEGG pathways in the transcriptomic comparisons; Table S15B: Selected enriched GO Biological Process terms in the transcriptomic comparisons.

## Abbreviations

Abbreviation	Full term
AKT	Protein kinase B
ALT	Alanine aminotransferase
ANOVA	Analysis of variance
AST	Aspartate aminotransferase
AUC0–t	Area under the curve from time zero to the last measurable concentration
AUC0–∞	Area under the curve from time zero extrapolated to infinity
BCA	Bicinchoninic acid
CLz/F	Apparent oral clearance
Cmax	Maximum observed plasma concentration
CYP2E1	Cytochrome P450 2E1
CYP3A	Cytochrome P450 3A
DAS	Drug and Statistics software
DEG	Differentially expressed gene
DLS	Dynamic light scattering
DMSO	Dimethyl sulfoxide
DPBS	Dulbecco’s phosphate-buffered saline
DSPC	1,2-distearoyl-sn-glycero-3-phosphocholine
EDTA	Ethylenediaminetetraacetic acid
EE	Encapsulation efficiency
FDR	False discovery rate
GAPDH	Glyceraldehyde-3-phosphate dehydrogenase
GO	Gene Ontology
GSEA	Gene set enrichment analysis
GSH	Reduced glutathione
H&E	Hematoxylin and eosin
HRP	Horseradish peroxidase
IACUC	Institutional Animal Care and Use Committee
KEGG	Kyoto Encyclopedia of Genes and Genomes
LC-MS/MS	Liquid chromatography-tandem mass spectrometry
LNP	Lipid nanoparticle
MDA	Malondialdehyde
MWCO	Molecular-weight cutoff
NAC	N-acetylcysteine
NCBI	National Center for Biotechnology Information
PDI	Polydispersity index
PI3K	Phosphoinositide 3-kinase
PK	Pharmacokinetics
p-AKT	Phosphorylated AKT
p-PI3K	Phosphorylated PI3K
PMSF	Phenylmethylsulfonyl fluoride
PVDF	Polyvinylidene fluoride
RIPA	Radioimmunoprecipitation assay
RNAi	RNA interference
ROS	Reactive oxygen species
RT-qPCR	Reverse transcription quantitative polymerase chain reaction
SD	Standard deviation
SDS-PAGE	Sodium dodecyl sulfate-polyacrylamide gel electrophoresis
si-*Cyp2e1*	Cyp2e1-targeting small interfering RNA
si-Control	Non-targeting control small interfering RNA
siRNA	Small interfering RNA
SOD	Superoxide dismutase
TEM	Transmission electron microscopy
Tmax	Time to reach maximum observed plasma concentration
TP	Triptolide
