# Supplementary material for: Liver-Directed Cyp2e1 RNA Interference Attenuates Hepatotoxicity Induced by Triptolide, a Bioactive Diterpenoid from Tripterygium wilfordii Hook. f
Source: Pharmaceuticals (Basel). 2026 Jul 15;19(7):1087. doi: 10.3390/ph19071087 (PMC13416182; doi:10.3390/ph19071087)
Supplement: Supplementary file 1 [file pharmaceuticals-19-01087-s001.zip › pharmaceuticals-4397457-supplementary.pdf]

Supplementary Material

Table S1. Compound information for the key chemicals used in this study.

Compound	Supplier	CAS No.	Purity	Catalog No.	Role in study / use
Triptolide (TP)	Must Bio-Technology Co., Ltd., Chengdu, China	38748-32-2	≥98% by HPLC	A0104	Hepatotoxic agent; used for TP-induced liver injury modeling
N-acetylcysteine (NAC)	Shanghai Yuanye Bio-Technology Co., Ltd., Shanghai, China	616-91-1	BR, 99%	S20137	Positive hepatoprotective control; 500 mg/kg/day by oral gavage in the subacute study
Finasteride	Shanghai Anpu-Cuishi Standards Technical Service Co., Ltd., Shanghai, China	154387-61-8	≥98.0%	T63539	LC-MS/MS internal standard

Table S2. Additional reagents and assay kits used in this study.

Reagent / kit	Supplier	Use / module
Dimethyl sulfoxide (DMSO)	Shanghai Macklin Biochemical Co., Ltd.	Preparation of TP stock solution
Normal saline	Wuhan Servicebio Technology Co., Ltd.	Vehicle and oral gavage dilution
BCA protein assay kit	GlpBio, USA	Protein quantification
ALT assay kit	Nanjing Jiancheng Bioengineering Institute	Serum biochemical analysis
AST assay kit	Nanjing Jiancheng Bioengineering Institute	Serum biochemical analysis
Tissue ROS assay kit	Shanghai BestBio Biotechnology Co., Ltd.	Hepatic oxidative stress assessment
Reduced glutathione (GSH) assay kit	Nanjing Jiancheng Bioengineering Institute	Hepatic antioxidant assessment
Malondialdehyde (MDA) assay kit	Nanjing Jiancheng Bioengineering Institute	Lipid peroxidation assessment
Superoxide dismutase (SOD) assay kit	Nanjing Jiancheng Bioengineering Institute	Antioxidant enzyme activity assessment
4% paraformaldehyde / neutral tissue fixative	Wuhan Servicebio Technology Co., Ltd.	Histological fixation
RNase remover	Sangon Biotech (Shanghai) Co., Ltd.	RNA-related procedures
Chemically modified siRNA duplexes	GenScript Biotech (Jiangsu, China)	siRNA synthesis for LNP loading
Anhydrous ethanol	Shanghai Macklin Biochemical Co., Ltd.	LNP lipid phase
DSPC	Shanghai Macklin Biochemical Co., Ltd.	LNP formulation lipid
High-purity cholesterol (CHO-HP)	AVT (Shanghai) Pharmaceutical Technology Co., Ltd.	LNP formulation lipid
DLin-MC3-DMA	AVT (Shanghai) Pharmaceutical Technology Co., Ltd.	LNP ionizable lipid
14:0 PEG2000 PE	Sigma, USA	PEGylated lipid in LNP formulation
DPBS	Sangon Biotech (Shanghai) Co., Ltd.	Dilution / injection buffer
Triton X-100	Biosharp	LNP disruption for RiboGreen assay
Quant-iT RiboGreen RNA assay kit	Thermo Fisher Scientific, USA	siRNA encapsulation-efficiency assay
RNase-free double-distilled water	Sangon Biotech (Shanghai) Co., Ltd.	Nuclease-free preparation
Acetic acid-sodium acetate buffer	Beijing Coolaber Technology Co., Ltd.	Aqueous phase for LNP preparation
Methanol	Sinopharm Chemical Reagent Co., Ltd.	LC-MS/MS sample and standard preparation
Ethyl acetate	Sinopharm Chemical Reagent Co., Ltd.	Plasma extraction for LC-MS/MS
EDTA	Zhenjiang Xiaomuchong Chemical Glass Instrument Co., Ltd.	Anticoagulant for plasma collection

Table S3. Major instruments and consumables used in this study.

Instrument / consumable	Supplier / manufacturer	Use / module
PZ-11AD-2 dual-channel precision syringe pump	Shanghai Pengzan Biotechnology Co., Ltd.	Microfluidic LNP preparation
Holder-3 LNP chip holder	Shanghai Pengzan Biotechnology Co., Ltd.	Microfluidic LNP preparation
Medium-volume LNP mixing chip	Shanghai Pengzan Biotechnology Co., Ltd.	Microfluidic LNP preparation
1 mL screw-tip syringe	BD, USA	Microfluidic input phase loading
3 mL screw-tip syringe	BD, USA	Microfluidic input phase loading
Dialysis bag	Spectrum, USA	Removal of residual ethanol after LNP preparation
PVDF membrane	Millipore, USA	Western blot transfer
NanoBrook 90Plus PALS instrument	Brookhaven Instruments, USA	Particle size, PDI, and zeta-potential measurement
Nanodrop spectrophotometer	Thermo Fisher Scientific, USA	RNA quality assessment
Transmission electron microscope	Hitachi, Japan	Nanoparticle morphology observation
Particle size analyzer	Brookhaven Instruments, USA	LNP physicochemical characterization
IKA T10 basic S25 tissue homogenizer	IKA, Germany	Tissue homogenization
Shimadzu LC-30AD chromatographic pump	Shimadzu, Japan	LC-MS/MS chromatographic system
SIL-30AC autosampler	Shimadzu, Japan	LC-MS/MS autosampling
CTO-20AC column oven	Shimadzu, Japan	LC-MS/MS column temperature control
ZORBAX SB-C18 column (3.0 × 100 mm, 3.5 μm)	Agilent Technologies	Chromatographic separation in LC-MS/MS analysis
Refrigerated centrifuge	Beckman, USA	Sample centrifugation
Rotary evaporator	Gongyi Yuxiang Instrument Co., Ltd.	Solvent evaporation during sample preparation
2 mL LC injection vials	Nalgene, USA	LC-MS/MS sample vials

Table S4. siRNA sequences used for Cyp2e1 knockdown and negative control.

Target	Sense strand (5′-3′)	Antisense strand (5′-3′)	Modification / purpose
Cyp2e1	ccAuGuAcAcAAuGGAAAAdTsdT	UUUUCcAUUGUGuAcAUGGdTsdT	Cyp2e1-targeting siRNA; 2′-O-methyl modification and dTsdT 3′ overhang
Negative control	cuuAcGcuGAGuAcuucGAdTsdT	UCGAAGuACUcAGCGuAAGdTsdT	Non-targeting control siRNA; 2′-O-methyl modification and dTsdT 3′ overhang

Table S5. Additional formulation, preparation, and characterization parameters of siRNA-loaded lipid nanoparticles.

Module	Parameter	Detail
Formulation	Lipid components	DLin-MC3-DMA, cholesterol, DSPC, and 14:0 PEG2000 PE
Formulation	Lipid molar ratio	50:38.5:10:1.5 (DLin-MC3-DMA:cholesterol:DSPC:14:0 PEG2000 PE)
Formulation	Aqueous phase	siRNA in 10 mM sodium acetate buffer
Mixing	Organic phase / aqueous phase volume ratio	1:3
Mixing	Device	Dual-channel syringe pump and LNP mixing chip
Purification	Dialysis	20 kDa dialysis bag; 2 h dialysis against RNase-free water in the dark to remove residual ethanol
Characterization	DLS dilution	LNP solution diluted with DPBS for physicochemical characterization; 100-fold dilution and ultrasonic dispersion were performed before measurement
Characterization	Particle size / PDI / zeta potential, si-Cyp2e1 LNPs	72.47 ± 1.15 nm; PDI 0.144 ± 0.008; −0.45 ± 0.10 mV
Characterization	Particle size / PDI / zeta potential, si-Control LNPs	71.26 ± 0.62 nm; PDI 0.213 ± 0.006; −0.37 ± 0.10 mV
Characterization	Morphology	Approximately spherical nanoparticles; about 80 nm by electron microscopy
Characterization	Encapsulation efficiency / drug loading, si-Cyp2e1 LNPs	82.89%; 0.46 mg/mL
Characterization	Encapsulation efficiency / drug loading, si-Control LNPs	75.31%; 0.45 mg/mL
Encapsulation assay	Calculation formula	EE% = [(total nucleic acid fluorescence − free nucleic acid fluorescence) / total nucleic acid fluorescence] × 100

Table S6. In vivo hepatic silencing validation design.

Group	Treatment	Dose	Endpoint / sample	Readout
Saline / DPBS control	Tail-vein injection of DPBS	Not applicable	Liver harvested 24 h after injection; n = 3	CYP2E1 protein expression by western blot
si-Control LNP	Tail-vein injection of si-Control LNPs	0.5 mg/kg siRNA equivalent	Liver harvested 24 h after injection; n = 3	CYP2E1 protein expression by western blot
si-Cyp2e1 LNP	Tail-vein injection of si-Cyp2e1 LNPs	0.5 mg/kg siRNA equivalent	Liver harvested 24 h after injection; n = 3	CYP2E1 protein expression by western blot

Table S7. Preliminary dose-titration experiments for establishing the repeated-exposure TP-induced subacute liver injury model.

Screening stage	TP dose	Duration	Biochemical response	Gross/histopathological response	Tolerability/mortality	Decision
First screening	250 µg/kg/day	14 days	Minimal or no obvious ALT/AST elevation	No obvious or minimal liver injury	Well tolerated; no excessive toxicity observed	Too mild for intervention analysis
First screening	500 µg/kg/day	14 days	Mild or unstable ALT/AST changes	Mild or inconsistent pathological injury	Well tolerated	Insufficiently stable injury
First screening	1000 µg/kg/day	Up to 14 days	Marked liver injury response	Severe pathological injury	Excessive toxicity/progressive mortality	Excluded as too severe
Second screening	600 µg/kg/day	7 days	Mild transaminase elevation	Mild liver injury	Acceptable tolerability	Not robust enough
Second screening	800 µg/kg/day	7 days	Reproducible ALT/AST elevation	Clear hepatocellular injury with inflammatory infiltration	Acceptable tolerability without excessive mortality	Selected for formal efficacy and mechanistic analyses

Note:The preliminary dose-titration experiments were used to guide model establishment. Dose selection was based on biochemical injury markers, gross liver appearance, H&E-confirmed histopathological changes, and tolerability/mortality. Corresponding dose-titration data for the 7-day 600 and 800 µg/kg/day regimens are shown in Supplementary Fig. S1. The formal efficacy and mechanistic studies were subsequently performed using TP at 800 µg/kg/day for 7 consecutive days.

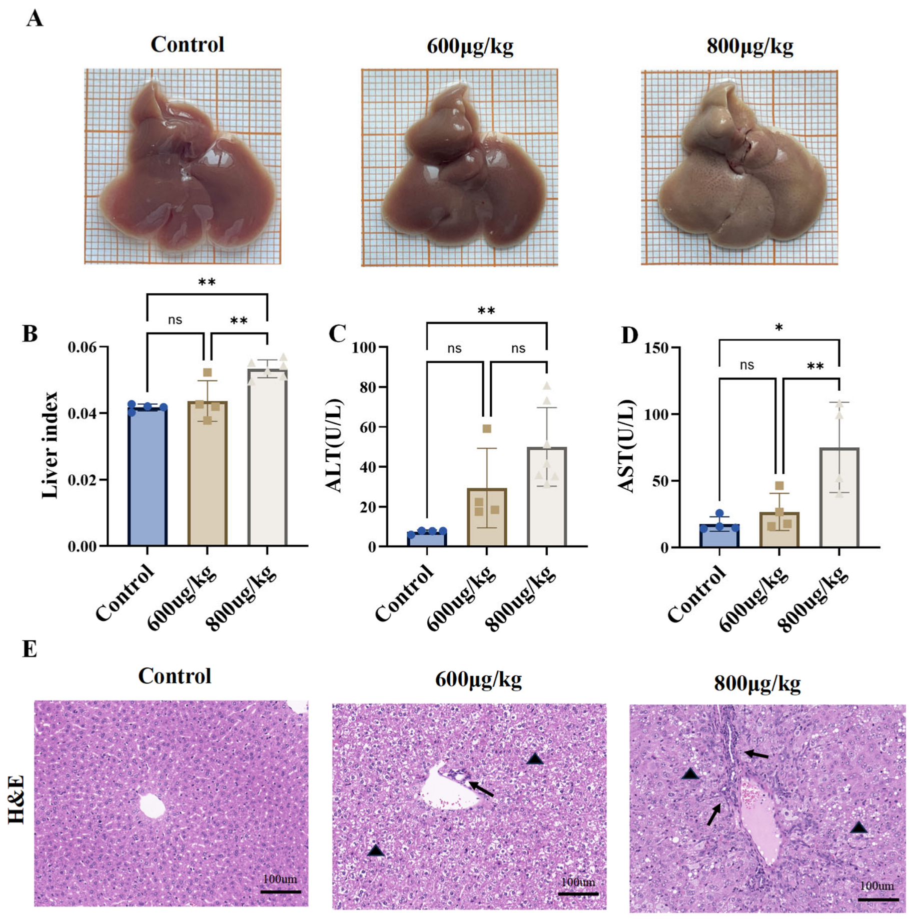

Figure S1. Dose-titration data supporting the selection of 800 μg/kg/day TP for 7 days as the subacute liver injury regimen. Female C57BL/6J mice were orally administered TP at 600 or 800 μg/kg/day for 7 consecutive days, and samples were collected 24 h after the last dose.

(A) Representative gross liver appearance.
(B) Liver index.
(C) Serum ALT levels.
(D) Serum AST levels.
(E) Representative H&E-stained liver sections.

Compared with the 600 μg/kg/day regimen, 800 μg/kg/day for 7 days produced more reproducible biochemical and histopathological liver injury while maintaining acceptable tolerability, and was therefore selected for the formal intervention study. Data are expressed as mean ± SD (n = 6 mice per group). Statistical analysis was performed by one-way ANOVA followed by Tukey's multiple-comparison test. *$P < 0.05$ and **$P < 0.01$ versus Control.

Table S8. Experimental grouping and treatment schedule for the subacute hepatoprotection study.

Group	TP administration	LNP / NAC intervention	Planned endpoint / initial group size
Control	Normal saline by oral gavage from day 4 to day 10	No TP; no protective intervention	Initially n = 6; endpoint analyses used available samples collected 24 h after the last scheduled dose
TP Model	TP 800 μg/kg/day by oral gavage from day 4 to day 10	No protective intervention	Initially n = 6; endpoint analyses used available samples collected on day 11
Pre-si-Control	TP 800 μg/kg/day by oral gavage from day 4 to day 10	si-Control LNPs by i.v. injection on days 1 and 7	Initially n = 6; endpoint analyses used available samples collected on day 11
Pre-si-Cyp2e1	TP 800 μg/kg/day by oral gavage from day 4 to day 10	si-Cyp2e1 LNPs by i.v. injection on days 1 and 7	Initially n = 6; endpoint analyses used available samples collected on day 11
si-Control	TP 800 μg/kg/day by oral gavage from day 4 to day 10	si-Control LNPs by i.v. injection on day 4	Initially n = 6; endpoint analyses used available samples collected on day 11
si-Cyp2e1	TP 800 μg/kg/day by oral gavage from day 4 to day 10	si-Cyp2e1 LNPs by i.v. injection on day 4	Initially n = 6; endpoint analyses used available samples collected on day 11
NAC	TP 800 μg/kg/day by oral gavage from day 4 to day 10	NAC 500 mg/kg/day by oral gavage from day 4 to day 10	Initially n = 6; endpoint analyses used available samples collected on day 11

Note: Mice were initially assigned at n = 6 per group. Because a small number of animals died before the scheduled endpoint, endpoint analyses were performed using available biological samples, and no artificial values were assigned for animals without evaluable samples. The actual number of biological replicates used for each endpoint is indicated by the plotted individual data points in the corresponding figures.

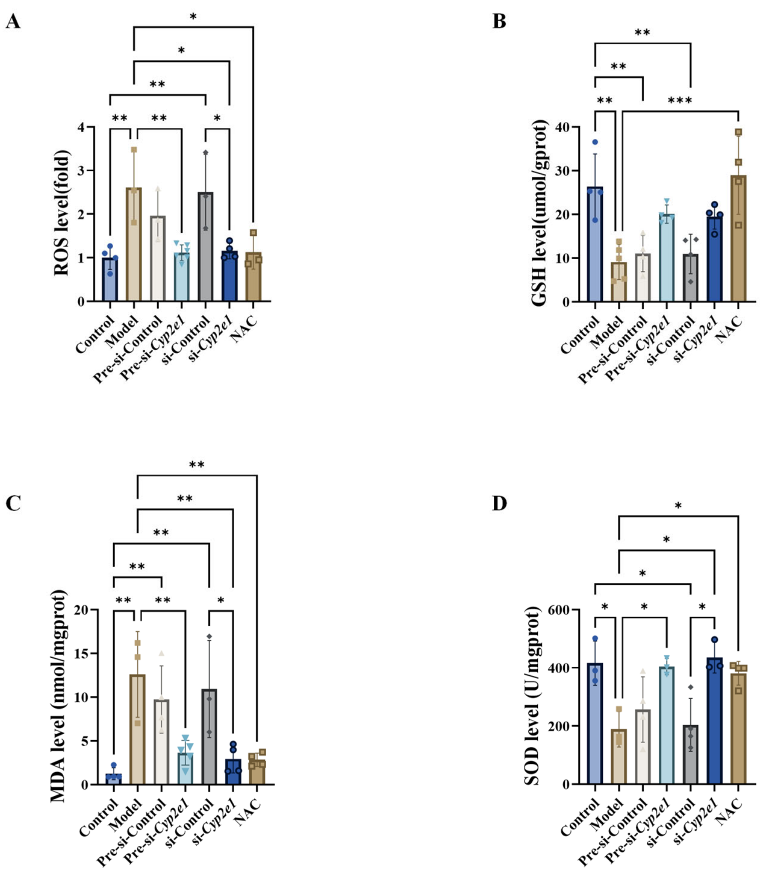

Figure S2. si-Cyp2e1 restored hepatic redox homeostasis in the TP-induced subacute liver injury model.
(A) Hepatic reactive oxygen species (ROS) levels.
(B) Hepatic reduced glutathione (GSH) levels.
(C) Hepatic malondialdehyde (MDA) levels.
(D) Hepatic superoxide dismutase (SOD) activity.
Data are expressed as mean ± SD (n = 4 endpoint-available biological samples per group). Statistical analysis was performed by one-way ANOVA followed by Tukey's multiple-comparison test. Statistical significance is indicated as follows: ns, not significant; *P < 0.05; **P < 0.01; ***P < 0.001.

Table S9. RNA sequencing design, quality control, and bioinformatic workflow.

Item	Detail
Sequenced groups	Control, TP Model, and si-Cyp2e1 LNP-treated groups
Biological replicates	Three biological replicates per group
Sample amount	Approximately 100 mg liver tissue per mouse
RNA extraction	TRIzol reagent
RNA quality criteria	RNA amount >2 μg; concentration >40 ng/μL; OD260/280 1.7–2.5; OD260/230 0.5–2.5; RIN >7
Sequencing platform	Illumina NovaSeq 6000, PE150 mode
Total sequencing output	50.57 G raw bases across 9 samples; 48.96 G valid bases after filtering
Clean-data quality summary	Valid read rate 96.53–96.90%; Q20 ≥99.99%; Q30 99.10–99.21%; GC content 49.0–49.5%
Reference-genome alignment summary	Total mapping rate 97.29–98.19%; uniquely mapped reads 89.81–91.92%; paired-end mapped reads 93.37–94.58%
Expression quantification	HTSeq and StringTie
Differential-expression screening	Limma; $P < 0.05$ and fold change >1.5 as exploratory criteria; adjusted P values were calculated using the Benjamini–Hochberg procedure
Multiple-testing correction	Benjamini–Hochberg correction was applied, and adjusted P values/FDR values are reported for selected GO and KEGG enrichment results in Tables S15A and S15B
Functional analyses	GO, KEGG, and GSEA; enrichment results were used for pathway-level hypothesis generation and downstream molecular validation

Table S10. RNA-seq sample-level data quality and alignment metrics.

Part A. Sequencing and clean-data quality metrics.

Sample	Raw reads	Valid reads	Valid (%)	Raw bases (G)	Valid bases (G)	GC (%)	Q20 (%)	Q30 (%)
Control-1	39,203,200	37,874,396	96.61	5.88	5.68	49.0	99.99	99.10
Control-2	37,301,042	36,131,338	96.86	5.60	5.42	49.5	100.00	99.21
Control-3	36,816,352	35,646,466	96.82	5.52	5.35	49.5	100.00	99.17
TP Model-1	41,132,704	39,851,910	96.89	6.17	5.98	49.0	100.00	99.20
TP Model-2	35,321,364	34,223,038	96.89	5.30	5.13	49.0	100.00	99.19
TP Model-3	38,928,556	37,720,984	96.90	5.84	5.66	49.5	100.00	99.18
si-Cyp2e1-1	36,430,176	35,274,904	96.83	5.46	5.29	49.5	100.00	99.20
si-Cyp2e1-2	37,830,606	36,517,134	96.53	5.67	5.48	49.5	100.00	99.20
si-Cyp2e1-3	34,227,046	33,119,054	96.76	5.13	4.97	49.0	100.00	99.12

Part B. Reference-genome alignment metrics.

Sample	Valid reads	Mapped reads (%)	Unique mapped reads (%)	Multi mapped reads (%)	PE mapped reads (%)
Control-1	37,874,396	36,975,514 (97.63)	34,095,941 (90.02)	2,879,573 (7.60)	35,420,972 (93.52)
Control-2	36,131,338	35,340,639 (97.81)	32,448,011 (89.81)	2,892,628 (8.01)	33,951,446 (93.97)
Control-3	35,646,466	34,678,815 (97.29)	32,086,496 (90.01)	2,592,319 (7.27)	33,325,526 (93.49)
TP Model-1	39,851,910	39,007,468 (97.88)	36,371,042 (91.27)	2,636,426 (6.62)	37,211,686 (93.37)
TP Model-2	34,223,038	33,568,097 (98.09)	31,431,032 (91.84)	2,137,065 (6.24)	32,270,722 (94.30)
TP Model-3	37,720,984	37,039,627 (98.19)	34,645,107 (91.85)	2,394,520 (6.35)	35,647,234 (94.50)
si-Cyp2e1-1	35,274,904	34,613,740 (98.13)	32,424,499 (91.92)	2,189,241 (6.21)	33,209,388 (94.14)
si-Cyp2e1-2	36,517,134	35,768,849 (97.95)	33,432,152 (91.55)	2,336,697 (6.40)	34,194,124 (93.64)
si-Cyp2e1-3	33,119,054	32,493,283 (98.11)	30,300,953 (91.49)	2,192,330 (6.62)	31,322,500 (94.58)

Note: Raw reads and raw bases refer to sequencing output before filtering. Valid reads and valid bases refer to clean data retained after quality filtering. Mapping statistics were calculated by aligning valid reads to the mouse reference genome. Values in parentheses indicate the corresponding percentage relative to valid reads.

Table S11. Primer sequences used for RT-qPCR validation.

Gene	Forward primer (5′-3′)	Reverse primer (5′-3′)	Application
Gapdh	AGGTCGGTGTGAACGGATTTG	TGTAGACCATGTAGTTGAGGTCA	Internal reference gene
Pi3k	ACCATCAGTGGCTCTGCGGTTT	GTGGTCTTCTGGGAACTCACCT	PI3K/AKT pathway validation
Akt	GGACTACTTGCACTCCGAGAAG	CATAGTGGCACCGTCCTTGATC	PI3K/AKT pathway validation
Il-6	TAGTCCTTCCTACCCCAATTTCC	TTGGTCCTTAGCCACTCCTTC	Inflammation-related validation
Il-1β	GCAACTGTTCCTGAACTCAACT	ATCTTTTGGGGTCCGTCAACT	Inflammation-related validation
Tnfα	ATGGCCTCCCTCTCATCAGT	CTTGGTGGTTTGCTACGACG	Inflammation-related validation

Table S12. RT-qPCR reaction system.

Component	Volume per 20 µL reaction
SYBR Green qPCR Mix (2×)	10.0 µL
Forward primer (10 µM)	0.4 µL
Reverse primer (10 µM)	0.4 µL
cDNA template	1.0 µL
Nuclease-free water	8.2 µL
Total	20.0 µL

Table S13. Primary antibodies used for western blot analysis.

Target / reagent	Antibody description	Catalog No.	Supplier	Use in study
CYP2E1	CYP2E1-specific monoclonal antibody	67263-1-Ig	Proteintech (Wuhan Sanying Biotechnology, Wuhan, China)	Hepatic CYP2E1 knockdown validation
GAPDH	GAPDH monoclonal antibody	60004-1-Ig	Proteintech (Wuhan Sanying Biotechnology, Wuhan, China)	Loading control
PI3K	PI3K antibody	21708-1-AP	Proteintech (Wuhan Sanying Biotechnology, Wuhan, China)	PI3K/AKT pathway validation
p-PI3K	Phospho-PI3K antibody	bs-5587R	Bioss Antibodies (Beijing, China)	PI3K phosphorylation validation
AKT	AKT monoclonal antibody	60203-2-Ig	Proteintech (Wuhan Sanying Biotechnology, Wuhan, China)	PI3K/AKT pathway validation
p-AKT	Phospho-AKT monoclonal antibody	66444-1-Ig	Proteintech (Wuhan Sanying Biotechnology, Wuhan, China)	AKT phosphorylation validation

Table S14. LC-MS/MS sample collection, calibration, and quantitative conditions.

**Module**	**Condition / parameter**	**Detail**
Animal preparation	Fasting	12 h before TP dosing, with free access to water
Blank plasma collection	Blank mouse plasma	Used as calibration matrix
PK groups in analysis	TP Model and si-Cyp2e1 LNP-treated groups	Three biologically independent plasma concentration-time profiles per group (n = 3 per group)
si-Cyp2e1 pretreatment	Timing	Tail-vein injection 72 h before TP administration
TP administration	Dose / route	800 μg/kg by oral gavage
Blood sampling points	Post-dose times	5, 10, 20, 30, and 45 min; 1, 1.5, 2, and 3 h
Plasma preparation	Centrifugation / storage	EDTA anticoagulation; 4000 rpm for 10 min; plasma stored at −80 °C
Internal standard	Finasteride solution	10 ng/mL
Sample extraction	Plasma / internal standard / solvent	100 μL plasma + 10 μL finasteride internal standard + 990 μL ethyl acetate
Sample reconstitution	Solvent / volume	60 μL of 80% methanol after evaporation to dryness at 45 °C
Calibration standards	Concentrations used for calibration	10, 20, 30, 50, 100, 200, 500, 750, and 1000 ng/mL
Retention time	Triptolide	3.72 ± 0.02 min
Retention time	Finasteride	5.10 ± 0.01 min
Solution calibration curve	Regression equation / R^2	$y = 2607.5x + 405.87$; $R^2 = 0.9992$
Plasma calibration curve	Regression equation / R^2	$y = 0.021x - 0.1106$; $R^2 = 0.9932$
Reported linear range / LLOQ	Range / lower limit	10–1000 ng/mL; lower limit of quantification, 10 ng/mL
Chromatography	Column	ZORBAX SB-C18 column (3.0 × 100 mm, 3.5 μm)
Chromatography	Column temperature / flow rate / injection volume	40 °C; 0.3 mL/min; 5 μL
Chromatography	Autosampler temperature	4 °C
Chromatography	Mobile phase / gradient	Methanol (A) and 0.05 mmol/L ammonium acetate (B); 0–3.4 min, 50–95% A; 3.4–3.9 min, 95% A; 3.9–4.5 min, 95–50% A; 4.5–7.0 min, 50% A
Mass spectrometry	Ionization / monitoring	Positive electrospray ionization mode with multiple reaction monitoring; finasteride used as internal standard

Table S15A. Selected enriched KEGG pathways in the transcriptomic comparisons.

Comparison	Pathway ID	KEGG pathway	Gene count	Rich factor	P value	Adjusted P value / FDR	Direction	Biological category	Representative genes
Control vs TP Model	mmu04151	PI3K-Akt signaling pathway	140	0.4192	7.65E-63	1.07E-60	Up-dominant	PI3K/AKT-associated signaling	Il6ra, Nos3, Ghr, Prlr, Erbb3, Mdm2, Col4a1, Lamc1, Thbs1, Itga6
Control vs TP Model	mmu04060	Cytokine-cytokine receptor interaction	94	0.3686	5.42E-37	1.16E-35	Up-dominant	Inflammation / immune response	Il1rap, Eda2r, Tnfrsf12a, Il6ra, Csf2rb, Bmp8b, Ghr, Lifr, Prlr, Cx3cr1
Control vs TP Model	mmu04666	Fc gamma R-mediated phagocytosis	56	0.5957	2.82E-36	5.36E-35	Up-dominant	Inflammation / immune response	Plpp1, Fcgr3, Limk1, Vav1, Marcks, Plpp2, Arf6, Inpp5d, Asap1, Gab2
Control vs TP Model	mmu04380	Osteoclast differentiation	65	0.4962	2.85E-35	4.87E-34	Up-dominant	Inflammation / immune response	Trem2, Fosl2, Itgb3, Fcgr3, Jun, Tgfbr2, Ncf2, Sirpb1c, Tnfrsf11b, Sirpa
Control vs TP Model	mmu04062	Chemokine signaling pathway	77	0.4162	8.27E-35	1.35E-33	Up-dominant	Inflammation / immune response	Cx3cr1, Ccl27a, Adcy1, Cxcl9, Ccr2, Ccl6, Cxcl10, Vav1, Rap1b, Grk5
Control vs TP Model	mmu04613	Neutrophil extracellular trap formation	76	0.4176	1.72E-34	2.64E-33	Up-dominant	Inflammation / immune response	C3, Selp, Actb, Hc, Hdac11, H4c8, Itgam, Itgb3, Fcgr3, C5ar1
Control vs TP Model	mmu04145	Phagosome	72	0.4390	1.77E-34	2.64E-33	Not available in source KEGG export	Inflammation / immune response	C3, H2-Q10, Tubb6, Atp6v0d2, Thbs1, Actb, C1rb, Itgam, Itgb3, Fcgr3
Control vs TP Model	mmu04670	Leukocyte transendothelial migration	56	0.5234	3.61E-32	4.57E-31	Not available in source KEGG export	Inflammation / immune response	Actb, Jam2, Msn, Myl12a, Ezr, Vcl, Itgam, Pecam1, Icam1, Actg1
Control vs TP Model	mmu04611	Platelet activation	56	0.4516	6.67E-28	5.43E-27	Not available in source KEGG export	Inflammation / immune response	Nos3, F2, Adcy1, Actb, Myl12a, Itgb3, Fcgr3, Actg1, Itga2, Col1a2
Control vs TP Model	mmu04610	Complement and coagulation cascades	48	0.5161	2.05E-27	1.49E-26	Not available in source KEGG export	Inflammation / immune response	C8g, C9, C2, Serpina1d, F7, F2, C3, Kng1, Serpina1c, Thbd
Control vs TP Model	mmu04621	NOD-like receptor signaling pathway	54	0.3333	1.64E-19	5.71E-19	Not available in source KEGG export	Inflammation / immune response	Gabarapl1, Trpv2, Gbp3, Jun, Il18, Sting1, Naip2, Ripk3, Itpr3, Gbp2b
Control vs TP Model	mmu04668	TNF signaling pathway	43	0.3675	1.24E-17	3.41E-17	Not available in source KEGG export	Inflammation / immune response	Lif, Jun, Icam1, Cxcl10, Ripk3, Map2k6, Fos, Rhbdf1, Vcam1, Mlkl
Control vs TP Model	mmu04620	Toll-like receptor signaling pathway	34	0.3736	1.70E-14	3.79E-14	Not available in source KEGG export	Inflammation / immune response	Cxcl9, Jun, Cd14, Cxcl10, Map2k6, Tlr1, Fos, Jak1, Pik3cd, Pik3r3
Control vs TP Model	mmu00590	Arachidonic acid metabolism	40	0.5000	2.07E-22	9.33E-22	Up-dominant	Oxidative stress / lipid peroxidation	Cyp2c54, Cyp2c70, Cyp2c50, Cyp2c23, Cyp2c29, Cyp2c40, Cyp2j5, Cyp2b13, Cyp2c37, Cbr3
Control vs TP Model	mmu00480	Glutathione metabolism	31	0.4366	1.41E-15	3.33E-15	Not available in source KEGG export	Oxidative stress / lipid peroxidation	Nat8f1, Gstt3, Gsta3, Gstt1, Odc1, Gpx3, Gsta1, Rrm1, G6pdx, Gstp1
Control vs TP Model	mmu05208	Chemical carcinogenesis - reactive oxygen species	50	0.2242	8.74E-11	1.53E-10	Not available in source KEGG export	Oxidative stress / lipid peroxidation	Cyp2f2, Gstt3, Gsta3, Cyp1a2, Gstt1, Cyp2e1, Gsta1, Jun, Ephx1, Cyp1b1

Comparison	Pathway ID	KEGG pathway	Gene count	Rich factor	P value	Adjusted P value / FDR	Direction	Biological category	Representative genes
Control vs TP Model	mmu04146	Peroxisome	24	0.2791	8.82E-08	1.24E-07	Not available in source KEGG export	Oxidative stress / lipid peroxidation	Paox, Nudt7, Pmvk, Pecr, Phyh, Pipox, Pxmp2, Baat, Acaa1b, Eci3
Control vs TP Model	mmu05417	Lipid and atherosclerosis	85	0.4337	5.87E-40	1.54E-38	Up-dominant	Xenobiotic / lipid metabolism	Cyp2j5, Nos3, Ldlr, Tnfrsf10b, Apoa4, Selp, Apaf1, Jun, Icam1, Bax
Control vs TP Model	mmu00140	Steroid hormone biosynthesis	56	0.5833	1.40E-35	2.51E-34	Down-dominant	Xenobiotic / lipid metabolism	Hsd17b6, Cyp2c54, Cyp2c70, Cyp2c50, Hsd11b1, Hsd17b8, Cyp2c23, Cyp2c29, Cyp17a1, Cyp2c40
Control vs TP Model	mmu00830	Retinol metabolism	47	0.4896	1.45E-25	9.00E-25	Down-dominant	Xenobiotic / lipid metabolism	Hsd17b6, Cyp2c54, Cyp2c70, Cyp2c50, Bco1, Cyp2c23, Cyp2c29, Cyp2c40, Rdh11, Cyp3a41a
Control vs TP Model	mmu00983	Drug metabolism - other enzymes	45	0.4891	1.62E-24	9.07E-24	Mixed	Xenobiotic / lipid metabolism	Ces1b, Ces1e, Ces1c, Dpyd, Ces1f, Gstt3, Tymp, Gsta3, Gstt1, Dpys
Control vs TP Model	mmu04976	Bile secretion	44	0.4583	1.49E-22	6.99E-22	Not available in source KEGG export	Xenobiotic / lipid metabolism	Sult2a8, Slc27a5, Slco1a1, Ldlr, Slc22a7, Abcb1b, Kcnn2, Abcc4, Sult2a1, Abcb11
Control vs TP Model	mmu00980	Metabolism of xenobiotics by cytochrome P450	35	0.4795	5.00E-19	1.68E-18	Not available in source KEGG export	Xenobiotic / lipid metabolism	Sult2a8, Cyp2f2, Hsd11b1, Dhdh, Cbr3, Gstt3, Sult2a1, Gsta3, Cyp1a2, Gstt1
Control vs TP Model	mmu00982	Drug metabolism - cytochrome P450	26	0.3881	7.31E-12	1.35E-11	Not available in source KEGG export	Xenobiotic / lipid metabolism	Fmo3, Maob, Gstt3, Gsta3, Cyp1a2, Gstt1, Ugt2b1, Cyp2e1, Ugt2a3, Gsta1
Control vs TP Model	mmu01040	Biosynthesis of unsaturated fatty acids	13	0.3824	1.60E-06	2.12E-06	Not available in source KEGG export	Xenobiotic / lipid metabolism	Scd1, Tecr, Fads1, Baat, Acaa1b, Acot2, Scd2, Hacd3, Hacd4, Elovl7
Control vs TP Model	mmu00071	Fatty acid degradation	15	0.3061	6.50E-06	8.35E-06	Not available in source KEGG export	Xenobiotic / lipid metabolism	Gcdh, Acat3, Acaa1b, Eci3, Cpt1c, Ehhadh, Hadhb, Acsl4, Aldh1b1, Cyp4a12b
Control vs TP Model	mmu04810	Regulation of actin cytoskeleton	104	0.4771	1.47E-53	1.25E-51	Up-dominant	ECM / cell adhesion remodeling	C8g, C9, F2, Kng1, Itga6, Iqgap1, Actb, Hc, Myh9, Msn
Control vs TP Model	mmu04820	Cytoskeleton in muscle cells	101	0.4591	4.40E-50	2.15E-48	Up-dominant	ECM / cell adhesion remodeling	Nid1, Lmna, Csrp3, Tmem43, Vim, Col5a2, Tpm4, Csrp1, Col4a1, Thbs1
Control vs TP Model	mmu04510	Focal adhesion	94	0.4796	8.97E-49	3.41E-47	Up-dominant	ECM / cell adhesion remodeling	Col4a1, Lamc1, Thbs1, Itga6, Flna, Vtn, Actb, Igf1, Col4a2, Lama5
Control vs TP Model	mmu04530	Tight junction	65	0.4037	1.13E-28	9.93E-28	Not available in source KEGG export	ECM / cell adhesion remodeling	Synpo, Actb, Myh9, Jam2, Msn, Myl12a, Ezr, Jun, Arhgef2, Actg1
Control vs TP Model	mmu04514	Cell adhesion molecules	59	0.3907	2.86E-25	1.75E-24	Not available in source KEGG export	ECM / cell adhesion remodeling	Cntnap1, H2-Q10, Nectin3, Selp, Itga6, Jam2, Cd34, Itgam, Pecam1, Sdc2
Control vs TP Model	mmu04540	Gap junction	41	0.5062	3.37E-23	1.67E-22	Not available in source KEGG export	ECM / cell adhesion remodeling	Tubb6, Adcy1, Gja1, Pdgfra, Tubb2a, Tuba8, Mapk7, Tubb3, Itpr3, Tubb2b
Control vs TP Model	mmu04512	ECM-receptor interaction	40	0.4706	3.67E-21	1.44E-20	Not available in source KEGG	ECM / cell adhesion remodeling	Col4a1, Lamc1, Thbs1, Itga6, Vtn, Col4a2, Lama5, Itgb3, Lamb3, Itga2

Comparison	Pathway ID	KEGG pathway	Gene count	Rich factor	P value	Adjusted P value / FDR	Direction	Biological category	Representative genes
							export		
Control vs TP Model	mmu04110	Cell cycle	84	0.5455	1.70E-49	7.25E-48	Up-dominant	Regulated cell death / cell cycle	Mcm2, Mdm2, Ccna2, Mcm6, Myc, Mcm5, Mcm3, Pcna, Mtbp, Aurkb
Control vs TP Model	mmu04210	Apoptosis	58	0.4361	7.41E-28	5.90E-27	Not available in source KEGG export	Regulated cell death / cell cycle	Lmna, Csf2rb, Tnfrsf10b, Csf2rb2, Actb, Apaf1, Jun, Actg1, Bax, Tuba8
Control vs TP Model	mmu04218	Cellular senescence	62	0.3690	6.77E-25	3.92E-24	Not available in source KEGG export	Regulated cell death / cell cycle	H2-Q10, Mdm2, Mapkapk2, Ccna2, Myc, Tgfbr2, Foxm1, Calm2, Slc25a4, Ccnb2
Control vs TP Model	mmu04115	p53 signaling pathway	38	0.5135	6.77E-22	2.97E-21	Not available in source KEGG export	Regulated cell death / cell cycle	Zmat3, Ccng1, Tnfrsf10b, Mdm2, Gtse1, Thbs1, Igf1, Apaf1, Bax, Ccnb2
Control vs TP Model	mmu04216	Ferroptosis	12	0.3000	6.76E-05	8.20E-05	Not available in source KEGG export	Regulated cell death / cell cycle	Trf, Inhca, Trp53, Hmox1, Acsl4, Cybb, Slc7a11, Gss, Sat1, Gclc
Control vs TP Model	mmu04010	MAPK signaling pathway	119	0.4146	6.30E-53	4.31E-51	Up-dominant	Stress signaling / signal transduction	Il1rap, Erbb3, Stmn1, Flna, Igf1, Kitl, Mapkapk2, Myc, Map3k6, Jun
Control vs TP Model	mmu04015	Rap1 signaling pathway	90	0.4455	2.03E-43	6.30E-42	Up-dominant	Stress signaling / signal transduction	Thbs1, Adcy1, Actb, Igf1, Kitl, Itgam, Itgb3, Actg1, Pdgfra, Rapgef5
Control vs TP Model	mmu04014	Ras signaling pathway	93	0.4227	1.85E-42	5.27E-41	Up-dominant	Stress signaling / signal transduction	Ets2, Igf1, Kitl, Pdgfra, Rapgef5, Vegfb, Calm2, Hgf, Rap1b, Arf6
Control vs TP Model	mmu04072	Phospholipase D signaling pathway	60	0.4348	1.11E-28	9.93E-28	Not available in source KEGG export	Stress signaling / signal transduction	F2, Plpp1, Adcy1, Kitl, Cyth4, Agt, Pdgfra, Arf2, Agpat5, Plpp2
Control vs TP Model	mmu04020	Calcium signaling pathway	76	0.3348	5.12E-27	3.58E-26	Not available in source KEGG export	Stress signaling / signal transduction	Nos3, Adra1b, Erbb3, Adcy1, Erbb4, Pdgfra, Cd38, Vegfb, Calm2, Hgf
Control vs TP Model	mmu04350	TGF-beta signaling pathway	51	0.4722	1.12E-26	7.51E-26	Not available in source KEGG export	Stress signaling / signal transduction	Hamp2, Trf, Bmp8b, Inhca, Tfr2, Thbs1, Fbn1, Myc, Inhbb, Tgfbr2
Control vs TP Model	mmu04024	cAMP signaling pathway	70	0.3518	1.84E-26	1.21E-25	Not available in source KEGG export	Stress signaling / signal transduction	Fxyd1, Gria3, Abcc4, Adcy1, Sucnr1, Pde4c, Jun, Atp1a3, Calm2, Vav1
Control vs TP Model	mmu04926	Relaxin signaling pathway	52	0.4228	2.39E-24	1.28E-23	Not available in source KEGG export	Stress signaling / signal transduction	Nos3, Col4a1, Adcy1, Col4a2, Jun, Tgfbr2, Col1a2, Vegfb, Tgfb1, Ednrb
Control vs TP Model	mmu04390	Hippo signaling pathway	58	0.3816	3.03E-24	1.59E-23	Not available in source KEGG export	Stress signaling / signal transduction	Bmp8b, Wnt9b, Actb, Ajuba, Myc, Tead1, Afp, Actg1, Tgfbr2, Ccn2
Control vs TP Model	mmu04068	FoxO signaling pathway	51	0.4048	7.25E-23	3.54E-22	Not available in source KEGG export	Stress signaling / signal transduction	Depp1, Gabarapl1, Mdm2, Homer2, Plk2, Igf1, G6pc1, Sgk1, Tgfbr2, Prmt1

Comparison	Pathway ID	KEGG pathway	Gene count	Rich factor	P value	Adjusted P value / FDR	Direction	Biological category	Representative genes
Control vs TP Model	mmu04012	ErbB signaling pathway	40	0.4878	6.77E-22	2.97E-21	Not available in source KEGG export	Stress signaling / signal transduction	Erbb3, Myc, Jun, Erbb4, Btc, Nrg1, Pik3cd, Pik3r3, Tgfa, Prkca
Control vs TP Model	mmu00120	Primary bile acid biosynthesis	9	0.5000	4.68E-06	6.06E-06	Not available in source KEGG export	Other enriched pathway	Slc27a5, Cyp27a1, Cyp7b1, Cyp39a1, Baat, Akr1d1, Cyp8b1, Ch25h, Cyp7a1
TP Model vs si-Cyp2e1	mmu04151	PI3K-Akt signaling pathway	93	0.2784	5.83E-46	9.76E-44	Down-dominant	PI3K/AKT-associated signaling	Lamc1, Nos3, Col1a1, Itgb3, Thbs1, Itga9, Col4a2, Itga6, Ghr, Erbb3
TP Model vs si-Cyp2e1	mmu04060	Cytokine-cytokine receptor interaction	69	0.2706	1.65E-33	6.92E-32	Down-dominant	Inflammation / immune response	Il1rap, Csf2rb2, Lif, Ghr, Il33, Cx3cr1, Tgfbr2, Prlr, Lifr, Tnfrsf11b
TP Model vs si-Cyp2e1	mmu04610	Complement and coagulation cascades	41	0.4409	5.92E-30	1.52E-28	Mixed	Inflammation / immune response	Thbd, Cfhr1, Mbl2, Plat, C9, F10, Vtn, C5ar1, Plg, C8g
TP Model vs si-Cyp2e1	mmu04670	Leukocyte transendothelial migration	41	0.3832	4.93E-27	1.10E-25	Down-dominant	Inflammation / immune response	Pecam1, Cldn4, Jam2, Vcam1, Cdh5, Msn, Ezr, Actg1, Itgam, Mmp2
TP Model vs si-Cyp2e1	mmu04611	Platelet activation	43	0.3468	3.28E-26	6.87E-25	Down-dominant	Inflammation / immune response	Nos3, Col1a1, Itgb3, Col3a1, Col1a2, Src, Fcgr3, Ptgs1, Actg1, Gucy1a1
TP Model vs si-Cyp2e1	mmu04750	Inflammatory mediator regulation of TRP channels	41	0.3445	6.26E-25	9.98E-24	Mixed	Inflammation / immune response	Cyp2c54, Cyp2j5, Cyp2c23, Il1rap, Cyp2c38, Cyp2c29, Cyp2c50, Cyp2c67, Cyp2c37, Cyp2c70
TP Model vs si-Cyp2e1	mmu04062	Chemokine signaling pathway	49	0.2649	1.01E-23	1.53E-22	Down-dominant	Inflammation / immune response	Src, Cx3cr1, Gng2, Arrb1, Ccr1, Vav1, Adcy1, Gnai1, Ncf1, Ccr5
TP Model vs si-Cyp2e1	mmu04640	Hematopoietic cell lineage	30	0.3297	5.31E-18	4.14E-17	Down-dominant	Inflammation / immune response	Itgb3, Itga6, Cd24a, Cd34, Cd14, Itga3, Kitl, Cd38, Itgam, Itga2
TP Model vs si-Cyp2e1	mmu04666	Fc gamma R-mediated phagocytosis	30	0.3191	1.49E-17	1.09E-16	Down-dominant	Inflammation / immune response	Plpp1, Gab2, Fcgr3, Inpp5d, Marcks, Vav1, Sphk1, Ncf1, Vasp, Wasf2
TP Model vs si-Cyp2e1	mmu04613	Neutrophil extracellular trap formation	40	0.2198	1.55E-16	9.13E-16	Down-dominant	Inflammation / immune response	Itgb3, Hdac11, H3c15, Slc25a4, Src, C5ar1, Fcgr3, Tlr8, Hc, Actg1
TP Model vs si-Cyp2e1	mmu04650	Natural killer cell mediated cytotoxicity	28	0.2772	1.02E-14	4.76E-14	Down-dominant	Inflammation / immune response	Vav1, Itgb2, Tnfrsf10b, Ifngr1, Vav3, Ppp3r1, Prkcg, Ppp3cc, Lcp2, Shc2
TP Model vs si-Cyp2e1	mmu04621	NOD-like receptor signaling pathway	33	0.2037	6.61E-13	2.43E-12	Down-dominant	Inflammation / immune response	Gabarapl1, Naip2, Trpv2, Oas2, Tlr4, Rnasel, P2rx7, Itpr3, Nlrp6, Sting1
TP Model vs si-Cyp2e1	mmu04668	TNF signaling pathway	26	0.2222	2.30E-11	6.88E-11	Down-dominant	Inflammation / immune response	Lif, Vcam1, Sele, Mmp14, Creb3l1, Akt3, Map2k4, Mapk13, Rhbdf1, Rps6ka4
TP Model vs si-Cyp2e1	mmu04662	B cell receptor signaling pathway	21	0.2658	5.19E-11	1.49E-10	Down-dominant	Inflammation / immune response	Inpp5d, Lilrb4b, Vav1, Pirb, Ifitm1, Akt3, Vav3, Lilrb4a, Ppp3r1, Ppp3cc
TP Model vs si-Cyp2e1	mmu04061	Viral protein interaction with cytokine and cytokine receptor	22	0.2500	6.76E-11	1.90E-10	Down-dominant	Inflammation / immune response	Cx3cr1, Ccr1, Csf1r, Ccr5, Il20rb, Tnfrsf10b, Cxcl14, Ccl6, Cxcl10, Cxcl5
TP Model vs si-Cyp2e1	mmu05323	Rheumatoid arthritis	20	0.2500	4.93E-10	1.21E-09	Down-dominant	Inflammation / immune response	Itgb2, Tgfb1, Tlr4, Tgfb3, Atp6v0e2, Tgfb2, Atp6v0a4, Atp6v0d2, Tnfrsf11a, Mmp3
TP Model vs si-Cyp2e1	mmu00983	Drug metabolism - other enzymes	29	0.3152	7.52E-17	4.84E-16	Up-dominant	Xenobiotic metabolism	Ces1e, Ces1b, Ces1f, Ces1c, Ugt2b1, Ugt2a3, Ces1d, Ces1a, Dpys, Dpyd
TP Model vs si-Cyp2e1	mmu00980	Metabolism of xenobiotics by cytochrome P450	25	0.3425	1.11E-15	5.72E-15	Up-dominant	Xenobiotic metabolism	Sult2a8, Cbr3, Adh4, Cyp2f2, Dhdh, Gstk1, Sult2a7, Ugt2b1, Ugt2a3, Cyp1b1

Comparison	Pathway ID	KEGG pathway	Gene count	Rich factor	P value	Adjusted P value / FDR	Direction	Biological category	Representative genes
TP Model vs si-Cyp2e1	mmu00982	Drug metabolism - cytochrome P450	21	0.3134	1.55E-12	5.57E-12	Up-dominant	Xenobiotic metabolism	Adh4, Maob, Gstk1, Ugt2b1, Ugt2a3, Gstm7, Ugt2b36, Gstt1, Gsta3, Aox3
TP Model vs si-Cyp2e1	mmu00480	Glutathione metabolism	19	0.2676	3.77E-10	9.49E-10	Mixed	Oxidative stress / redox metabolism	Nat8f1, Gpx3, Gstk1, Gstm7, Gstt1, Gsta3, Pgd, Gstm6, Hpgds, Nat8f2
TP Model vs si-Cyp2e1	mmu05208	Chemical carcinogenesis - reactive oxygen species	33	0.1480	4.40E-09	9.97E-09	Down-dominant	Oxidative stress / redox metabolism	Cyp2f2, Slc25a4, Src, Prkd3, Cyp1b1, Gstm7, Hgf, Gstt1, Gsta3, Gstm6
TP Model vs si-Cyp2e1	mmu04146	Peroxisome	17	0.1977	3.91E-07	7.31E-07	Up-dominant	Oxidative stress / redox metabolism	Nudt7, Pecr, Phyh, Gstk1, Pipox, Slc27a2, Pxmp2, Agxt, Acaa1b, Hao1
TP Model vs si-Cyp2e1	mmu00140	Steroid hormone biosynthesis	45	0.4688	3.26E-34	1.56E-32	Up-dominant	Lipid / steroid / bile metabolism	Sult1e1, Cyp2c54, Cyp2c23, Hsd3b2, Cyp2c38, Cyp7b1, Hsd17b6, Cyp2c29, Cyp2c50, Hsd3b3
TP Model vs si-Cyp2e1	mmu00830	Retinol metabolism	36	0.3750	1.59E-23	2.31E-22	Up-dominant	Lipid / steroid / bile metabolism	Cyp2c54, Cyp2c23, Cyp2c38, Hsd17b6, Cyp2c29, Cyp2c50, Adh4, Cyp2c67, Cyp2c37, Cyp2c70
TP Model vs si-Cyp2e1	mmu04976	Bile secretion	30	0.3125	2.90E-17	1.94E-16	Mixed	Lipid / steroid / bile metabolism	Sult2a8, Kcnn2, Slc27a5, Slc22a7, Ldlr, Sult2a7, Ugt2b1, Abcb11, Aqp1, Ugt2a3
TP Model vs si-Cyp2e1	mmu00590	Arachidonic acid metabolism	27	0.3375	1.23E-16	7.33E-16	Mixed	Lipid / steroid / bile metabolism	Cyp2c54, Cyp2j5, Cyp2c23, Cyp2c38, Cyp2c29, Cbr3, Cyp2c50, Cyp2c67, Cyp2c37, Cyp2c70
TP Model vs si-Cyp2e1	mmu00591	Linoleic acid metabolism	20	0.4000	2.50E-14	1.07E-13	Up-dominant	Lipid / steroid / bile metabolism	Cyp2c54, Cyp2j5, Cyp2c23, Cyp2c38, Cyp2c29, Cyp2c50, Cyp2c67, Cyp2c37, Cyp2c70, Cyp2c68
TP Model vs si-Cyp2e1	mmu04913	Ovarian steroidogenesis	17	0.3091	2.63E-10	6.89E-10	Mixed	Lipid / steroid / bile metabolism	Cyp2j5, Hsd3b2, Hsd3b3, Ldlr, Cyp1b1, Hsd17b2, Igf1, Adcy1, Cyp2j9, Igf1r
TP Model vs si-Cyp2e1	mmu00600	Sphingolipid metabolism	14	0.2745	5.41E-08	1.10E-07	Down-dominant	Lipid / steroid / bile metabolism	Acer2, Cerk, B4galt6, Plpp1, Degs1l, Sgms1, Cers5, Sphk1, Galc, Sptlc2
TP Model vs si-Cyp2e1	mmu04979	Cholesterol metabolism	13	0.2600	3.24E-07	6.14E-07	Mixed	Lipid / steroid / bile metabolism	Lipc, Apoa2, Ldlr, Abcb11, Angptl3, Apoc1, Soat1, Cideb, Mylip, Osbpl5
TP Model vs si-Cyp2e1	mmu00565	Ether lipid metabolism	12	0.2553	1.14E-06	2.04E-06	Down-dominant	Lipid / steroid / bile metabolism	Pla2g7, Plpp1, Tmem86b, Peds1, Lpcat1, Gdpd1, Agps, Pld2, Pla2g4a, Lpcat2
TP Model vs si-Cyp2e1	mmu00100	Steroid biosynthesis	8	0.4000	1.65E-06	2.90E-06	Up-dominant	Lipid / steroid / bile metabolism	Sc5d, Ebp, Soat1, Fdft1, Cyp51, Cyp2r1, Tm7sf2, Nsdhl
TP Model vs si-Cyp2e1	mmu04820	Cytoskeleton in muscle cells	76	0.3455	3.28E-45	3.67E-43	Down-dominant	ECM / cell adhesion remodeling	Col5a2, Csrp3, Col5a1, Fbn1, Col1a1, Nid1, Itgb3, Col3a1, Thbs1, Itga9
TP Model vs si-Cyp2e1	mmu04510	Focal adhesion	70	0.3571	7.70E-43	6.45E-41	Down-dominant	ECM / cell adhesion remodeling	Emp1, Lamc1, Col1a1, Itgb3, Thbs1, Itga9, Col4a2, Itga6, Col6a1, Col1a2
TP Model vs si-Cyp2e1	mmu04810	Regulation of actin cytoskeleton	62	0.2844	1.39E-31	4.24E-30	Down-dominant	ECM / cell adhesion remodeling	Itgb3, Itga9, Iqgap1, Itga6, C9, Itga3, Src, Pdgfra, Msn, C8g
TP Model vs si-Cyp2e1	mmu04512	ECM-receptor interaction	33	0.3882	3.03E-22	3.63E-21	Down-dominant	ECM / cell adhesion remodeling	Lamc1, Col1a1, Itgb3, Thbs1, Itga9, Hspg2, Col4a2, Itga6, Col6a1, Col1a2
TP Model vs si-Cyp2e1	mmu04514	Cell adhesion molecules	40	0.2649	1.26E-19	1.21E-18	Down-dominant	ECM / cell adhesion remodeling	Pecam1, Itga9, Itga6, Cldn4, Jam2, Cd34, Vcam1, Cdh5, Sdc2, Vsir
TP Model vs si-Cyp2e1	mmu04148	Efferocytosis	48	0.3019	5.02E-26	9.90E-25	Down-dominant	Regulated cell death / clearance	Pecam1, Itgb3, Thbs1, Cd24a, Ano6, Sgk1, Mfge8, Cx3cr1, Ch25h, Arg2
TP Model vs si-Cyp2e1	mmu04210	Apoptosis	27	0.2030	8.75E-11	2.42E-10	Down-dominant	Regulated cell death / clearance	Csf2rb2, Apaf1, Actg1, Ctss, Ngf, Itpr3, Tuba1a, Bcl2l11, Tnfrsf10b, Csf2rb

Comparison	Pathway ID	KEGG pathway	Gene count	Rich factor	P value	Adjusted P value / FDR	Direction	Biological category	Representative genes
TP Model vs si-Cyp2e1	mmu04217	Necroptosis	22	0.1486	1.47E-06	2.62E-06	Down-dominant	Regulated cell death / clearance	Zbp1, Slc25a4, Il33, Tlr4, Spata2l, Chmp4c, Camk2d, Tnfrsf10b, Pygm, Cybb
TP Model vs si-Cyp2e1	mmu04140	Autophagy - animal	16	0.1000	3.35E-03	4.38E-03	Down-dominant	Regulated cell death / clearance	Gabarapl1, Deptor, Ddit4, Rraga, Akt3, Hif1a, Bcl2l1, Igf1r, Nras, Ppp2cb
TP Model vs si-Cyp2e1	mmu04215	Apoptosis - multiple species	5	0.1613	1.35E-02	1.64E-02	Down-dominant	Regulated cell death / clearance	Apaf1, Bcl2l11, Ngfr, Bcl2l1, Bak1
TP Model vs si-Cyp2e1	mmu04015	Rap1 signaling pathway	63	0.3119	1.12E-34	6.26E-33	Down-dominant	Stress signaling / signal transduction	Itgb3, Thbs1, Src, Rapgef5, Pdgfra, Prkd3, Kitl, Hgf, Actg1, Itgam
TP Model vs si-Cyp2e1	mmu04010	MAPK signaling pathway	73	0.2544	1.89E-33	7.04E-32	Down-dominant	Stress signaling / signal transduction	Hspa1b, Il1rap, Hspa1a, Erbb3, Map4k4, Cd14, Pdgfra, Tgfbr2, Map3k6, Kitl
TP Model vs si-Cyp2e1	mmu04020	Calcium signaling pathway	60	0.2643	1.08E-28	2.57E-27	Down-dominant	Stress signaling / signal transduction	Adra1b, Nos3, Erbb3, Slc25a4, Ednrb, Pdgfra, Ednra, Orai2, Slc8a1, Hgf
TP Model vs si-Cyp2e1	mmu04014	Ras signaling pathway	55	0.2500	4.10E-25	6.86E-24	Down-dominant	Stress signaling / signal transduction	Rapgef5, Gab2, Pdgfra, Kitl, Hgf, Gng2, Fgfr2, Igf1, Ets1, Csf1r
TP Model vs si-Cyp2e1	mmu04024	cAMP signaling pathway	48	0.2412	2.24E-21	2.51E-20	Down-dominant	Stress signaling / signal transduction	Pde4c, Abcc4, Fxyd1, Ednra, Sucnr1, Vav1, Hcar2, Gabbr2, Adcy1, Gnai1
TP Model vs si-Cyp2e1	mmu04022	cGMP-PKG signaling pathway	43	0.2638	6.47E-21	6.77E-20	Down-dominant	Stress signaling / signal transduction	Adra1b, Nos3, Slc25a4, Ednrb, Ednra, Slc8a1, Adrb3, Kcnj8, Gucy1a1, Prkg1
TP Model vs si-Cyp2e1	mmu05204	Chemical carcinogenesis - DNA adducts	33	0.3882	3.03E-22	3.63E-21	Up-dominant	Xenobiotic / oxidative injury-related pathway	Sult2a8, Cyp2c54, Cyp2c23, Cyp2c38, Cyp2c29, Cyp2c50, Cyp2c67, Cyp2c37, Cyp2c70, Gstk1
TP Model vs si-Cyp2e1	mmu05207	Chemical carcinogenesis - receptor activation	46	0.2190	9.66E-19	8.30E-18	Mixed	Xenobiotic / oxidative injury-related pathway	Ugt2b1, Klf4, Ugt2a3, Src, Cyp2b13, Cyp1b1, Gstm7, Ugt2b36, Adrb3, Gstt1

Note: This table summarizes selected enriched KEGG pathways from the two RNA-seq comparisons. The enrichment results were used for pathway-level interpretation and hypothesis generation. PI3K/AKT signaling was prioritized for downstream validation in the present study, whereas other enriched pathways require further targeted validation. Ferroptosis-related KEGG enrichment was not interpreted as a primary validated mechanism because dedicated ferroptosis markers were not assessed.

Table S15B. Selected enriched GO Biological Process terms in the transcriptomic comparisons.

Comparison	GO ID	GO biological process	Gene count	Rich factor	P value	Adjusted P value / FDR	Direction	Biological category	Representative genes
Control vs TP Model	GO:0045087	innate immune response	169	0.3781	9.55E-24	2.04E-21	Predominantly upregulated	Inflammation / immune response	Tmem43, Adam15, Cx3cr1, Ifi27l2b, Trem2, Hmgb2, Fosl2, Pspc1, Tlr13, Arhgef2
Control vs TP Model	GO:0006954	inflammatory response	135	0.3913	8.67E-21	1.42E-18	Predominantly upregulated	Inflammation / immune response	Cx3cr1, Pla2g7, Selp, Thbs1, Hmgb2, Mapkapk2, Fosl2, Ccr2, Tlr13, C5ar1
Control vs TP Model	GO:0002376	immune system process	193	0.3277	1.09E-18	1.46E-16	Predominantly upregulated	Inflammation / immune response	Ctps1, Tmem43, Cx3cr1, Ifi27l2b, Hmgb2, Pspc1, Tlr13, Arhgef2, Cd14, Arid5a
Control vs TP Model	GO:0032496	response to lipopolysaccharide	75	0.4213	3.72E-14	3.30E-12	Predominantly upregulated	Inflammation / immune response	Nos3, Thbd, Gja1, Hmgb2, Mapkapk2, Fosl2, Jun, Alpl, Cd14, Akap12
Control vs TP Model	GO:0050729	positive regulation of inflammatory response	42	0.5185	4.16E-12	2.89E-10	Predominantly upregulated	Inflammation / immune response	Il17rb, Ccr2, Fabp4, Gprc5b, Il33, Adam8, App, Lgals1, Tgfb1, Ctss
Control vs TP Model	GO:0034097	response to cytokine	37	0.5000	3.04E-10	1.61E-08	Predominantly upregulated	Inflammation / immune response	Selp, Mapkapk2, Jun, Cd38, Coro1a, Fos, Timp1, Cxcl16, Skil, Fosl1
Control vs TP Model	GO:0043123	positive regulation of canonical NF-kappaB signal transduction	63	0.3865	3.11E-10	1.64E-08	Predominantly upregulated	Inflammation / immune response	Eda2r, Cx3cr1, Flna, Tgm2, Ajuba, Gprc5b, Ddx21, Trim6, Ednra, Cd36
Control vs TP Model	GO:0071222	cellular response to lipopolysaccharide	72	0.3445	6.64E-09	2.99E-07	Predominantly upregulated	Inflammation / immune response	Cx3cr1, Vim, Hmgb2, Ncl, Cd14, Arid5a, Gstp1, Sirpa, Cd36, Abcb1a
Control vs TP Model	GO:0070301	cellular response to hydrogen peroxide	38	0.5000	1.75E-10	9.77E-09	Predominantly upregulated	Oxidative stress / lipid peroxidation	Akr1b1, Mdm2, Klf4, Anxa1, Cyp1b1, Pcna, Smpd3, Aqp1, Mapk7, Sirpa
Control vs TP Model	GO:2000379	positive regulation of reactive oxygen species metabolic process	21	0.5385	4.21E-07	1.31E-05	Predominantly upregulated	Oxidative stress / lipid peroxidation	Thbs1, Tgfbr2, Akr1c18, Cyp1b1, Nfe2l2, Cd36, Cbr1, Ripk3, Trp53, Lcn2
Control vs TP Model	GO:0006979	response to oxidative stress	48	0.3429	2.36E-06	6.27E-05	Predominantly upregulated	Oxidative stress / lipid peroxidation	Srxn1, Cygb, Gpx3, Rcan1, Pxdn, Aldh3b1, G6pdx, Pcna, Ptgs1, App
Control vs TP Model	GO:0034599	cellular response to oxidative stress	34	0.3736	8.34E-06	1.88E-04	Predominantly upregulated	Oxidative stress / lipid peroxidation	Slc25a24, Selenon, Aldh3b1, G6pdx, Ermp1, Slc4a11, Ednra, Nfe2l2, Cd36, Nqo1
Control vs TP Model	GO:0072593	reactive oxygen species metabolic process	19	0.4419	5.79E-05	1.00E-03	Predominantly upregulated	Oxidative stress / lipid peroxidation	Ucp2, Ccn2, Vav1, Col6a1, Ripk3, Trp53, Sesn2, Cyba, P2rx7, Tigar
Control vs TP Model	GO:0034775	glutathione transmembrane transport	5	0.8333	9.29E-04	9.42E-03	Predominantly upregulated	Oxidative stress / lipid peroxidation	Abcc4, Slc13a3, Gja1, Slc7a11, Abcc1
Control vs TP Model	GO:0006801	superoxide metabolic process	10	0.5000	1.05E-03	9.99E-03	Predominantly upregulated	Oxidative stress / lipid peroxidation	Sh3pxd2b, Ncf2, Nqo1, Cyba, Cybb, Nrros, Nos2, Slc1a1
Control vs TP Model	GO:0006629	lipid metabolic process	284	0.4017	6.09E-45	3.71E-42	Mixed up/down	Xenobiotic / lipid metabolism	Bco1, Akr1b1, Tmem43, Pla2g7, Plpp1, Isyna1, Cerk, Acot2, Pam, Apoc2
Control vs TP Model	GO:0008202	steroid metabolic process	70	0.5263	1.13E-19	1.61E-17	Predominantly downregulated	Xenobiotic / lipid metabolism	Sult2a8, Hsd17b6, Fdps, Sult2a2, Insig1, Sc5d, Tm7sf2, Cyp27a1, Ebp, Nsdhl
Control vs TP Model	GO:0009410	response to xenobiotic stimulus	110	0.3780	5.92E-16	6.46E-14	Predominantly upregulated	Xenobiotic / lipid metabolism	Abcb1b, Mdm2, Thbs1, Adcy1, Pam, Fosl2, Jun, Nckap1l, Tgfbr2, Itga2
Control vs TP Model	GO:0008203	cholesterol metabolic process	57	0.5135	1.15E-15	1.23E-13	Mixed up/down	Xenobiotic / lipid metabolism	Sult2a8, Fdps, Lipc, Insig1, Apoc1, Tm7sf2, Apoa2, Cyp27a1, Ebp, Pon1
Control vs TP Model	GO:0006805	xenobiotic metabolic process	50	0.5319	1.03E-14	9.83E-13	Mixed up/down	Xenobiotic / lipid metabolism	Sult2a8, Cyp2c54, Fmo3, Cyp2f2, Cyp2c70, Cyp2c50, Cyp2c23, Cyp2c29, Cyp2c40, Cyp2j5
Control vs TP	GO:000663	fatty acid metabolic process	73	0.3822	2.36E-11	1.52E-09	Mixed up/down	Xenobiotic / lipid	Acot2, Cpt1c, Fabp4, Abhd5, Scd2, Lpin3, Cyp1b1,

Comparison	GO ID	GO biological process	Gene count	Rich factor	P value	Adjusted P value / FDR	Direction	Biological category	Representative genes
Model	1							metabolism	Ehhadh, Ptgs1, Alox12
Control vs TP Model	GO:0071466	cellular response to xenobiotic stimulus	36	0.4737	3.47E-09	1.63E-07	Predominantly upregulated	Xenobiotic / lipid metabolism	Eftud2, Itgb3, Myc, Acer2, Pcna, Nfe2l2, Rap1b, Trp53, Ppp1r9b, Uchl1
Control vs TP Model	GO:0019373	epoxygenase P450 pathway	20	0.6452	1.18E-08	5.15E-07	Predominantly downregulated	Xenobiotic / lipid metabolism	Cyp2c54, Cyp2f2, Cyp2c70, Cyp2c50, Cyp2c23, Cyp2c29, Cyp2c40, Cyp2j5, Cyp2b13, Cyp2c37
Control vs TP Model	GO:0042632	cholesterol homeostasis	39	0.4149	7.61E-08	2.80E-06	Mixed up/down	Xenobiotic / lipid metabolism	Lipc, Insig1, Apoa2, Ces1b, Ces1e, Scd1, Ces1c, Nr1d1, Angptl3, Ces1f
Control vs TP Model	GO:0007155	cell adhesion	214	0.4007	6.41E-34	2.69E-31	Predominantly upregulated	ECM / cell adhesion remodeling	Cd93, Nid1, Tnfrsf12a, Anxa2, Adam15, Cx3cr1, Cntnap1, Col12a1, Lamc1, Selp
Control vs TP Model	GO:0098609	cell-cell adhesion	79	0.4225	6.60E-15	6.46E-13	Predominantly upregulated	ECM / cell adhesion remodeling	Cd93, Cx3cr1, Selp, Fat1, Itga6, Lama5, Myh9, Jam2, Cd34, Pecam1
Control vs TP Model	GO:0030198	extracellular matrix organization	68	0.4416	3.65E-14	3.26E-12	Predominantly upregulated	ECM / cell adhesion remodeling	Nid1, Adamts1, Col5a2, Col4a1, Ccdc80, Emilin1, Matn2, Adamts15, Col4a2, Pxdn
Control vs TP Model	GO:0007229	integrin-mediated signaling pathway	44	0.5000	6.54E-12	4.48E-10	Predominantly upregulated	ECM / cell adhesion remodeling	Adam15, Itga6, Lama5, Itgam, Itgb3, Cdh17, Fyb2, Itga2, Ccn2, Plek
Control vs TP Model	GO:0007160	cell-matrix adhesion	39	0.4432	8.40E-09	3.73E-07	Predominantly upregulated	ECM / cell adhesion remodeling	Nid1, Anxa2, Emilin1, Itga6, Bcam, Cd34, Itgb3, Cd63, Itga2, Adam8
Control vs TP Model	GO:0030199	collagen fibril organization	28	0.5185	1.54E-08	6.57E-07	Predominantly upregulated	ECM / cell adhesion remodeling	Anxa2, Loxl2, Col5a2, Fkbp10, Pxdn, Col5a1, Cyp1b1, Col1a2, Col6a1, Lox
Control vs TP Model	GO:0051897	positive regulation of phosphatidylinositol 3-kinase/protein kinase B signal transduction	86	0.4599	6.95E-19	9.43E-17	Predominantly upregulated	PI3K/AKT-associated signaling	Cx3cr1, Unc5b, Selp, Thbs1, Trem2, Pdgfra, Akr1c18, Nrg1, Adam8, Hgf
Control vs TP Model	GO:0043491	phosphatidylinositol 3-kinase/protein kinase B signal transduction	33	0.3837	5.80E-06	1.36E-04	Predominantly upregulated	PI3K/AKT-associated signaling	Smpd3, Nrg1, Prex2, Plekha1, Col6a1, Btbd10, Col6a2, Lox, Sesn2, Cd2ap
Control vs TP Model	GO:0043065	positive regulation of apoptotic process	159	0.4344	3.60E-30	1.27E-27	Predominantly upregulated	Regulated cell death / apoptotic process	Phlda3, Tnfrsf12a, Nos3, Tnfrsf10b, Emilin1, Itga6, Frzb, Ano6, Tgm2, Igf2r
Control vs TP Model	GO:0006915	apoptotic process	217	0.3617	4.50E-27	1.24E-24	Predominantly upregulated	Regulated cell death / apoptotic process	Cidec, Phlda3, Tnfrsf12a, Aen, Mcm2, Tnfrsf10b, Ifi27l2b, Mdm2, Ckap2, Tpx2
Control vs TP Model	GO:0043066	negative regulation of apoptotic process	195	0.3686	1.29E-25	3.05E-23	Predominantly upregulated	Regulated cell death / apoptotic process	Akr1b1, Tnfrsf23, Ccng1, Mdm2, Plaur, Fstl1, Plk2, Thbs1, Flna, Ucp2
Control vs TP Model	GO:0042981	regulation of apoptotic process	77	0.3702	4.22E-11	2.60E-09	Predominantly upregulated	Regulated cell death / apoptotic process	Cidec, Tnfrsf10b, Actb, Tgm2, Gja1, Apaf1, Igf2r, Myc, Bax, Acer2
TP Model vs si-Cyp2e1	GO:0007155	cell adhesion	159	0.2978	4.45E-40	3.12E-37	Predominantly downregulated	ECM / cell adhesion remodeling	Cd93, Pcdh17, Pxdn, Col5a1, Gpnmb, Adam15, Lamc1, Nid1
TP Model vs si-Cyp2e1	GO:0030198	extracellular matrix organization	62	0.4026	4.17E-24	1.10E-21	Predominantly downregulated	ECM / cell adhesion remodeling	Col5a2, Ccdc80, Mmp7, Pxdn, Matn2, Col1a1, Nid1, Emilin1
TP Model vs si-Cyp2e1	GO:0007160	cell-matrix adhesion	35	0.3977	4.70E-14	4.84E-12	Predominantly downregulated	ECM / cell adhesion remodeling	Cd63, Nid1, Itgb3, Ccn2, Emilin1, Col3a1, Itga9, Adamts12
TP Model vs si-Cyp2e1	GO:0030199	collagen fibril organization	25	0.4630	3.28E-12	2.83E-10	Predominantly downregulated	ECM / cell adhesion remodeling	Loxl2, Col5a2, Pxdn, Col5a1, Fkbp10, Col1a1, Col3a1, Adamts2

Comparison	GO ID	GO biological process	Gene count	Rich factor	P value	Adjusted P value / FDR	Direction	Biological category	Representative genes
TP Model vs si-Cyp2e1	GO:0098609	cell-cell adhesion	50	0.2674	1.30E-11	1.02E-09	Predominantly downregulated	ECM / cell adhesion remodeling	Cd93, Pecam1, Fblim1, Itga9, Itga6, Jam2, Cd34, Kirrel1
TP Model vs si-Cyp2e1	GO:0022617	extracellular matrix disassembly	8	0.5333	2.44E-05	4.64E-04	Mixed	ECM / cell adhesion remodeling	Lamc1, Sh3pxd2b, Plg, Adamts15, Eng, Pbxip1, Mmp13, Exoc8
TP Model vs si-Cyp2e1	GO:0085029	extracellular matrix assembly	8	0.5333	2.44E-05	4.64E-04	Predominantly downregulated	ECM / cell adhesion remodeling	Fkbp10, Col6a1, Col1a2, Tgfb1, Eln, Smpd3, Sox9, Gpm6b
TP Model vs si-Cyp2e1	GO:0045087	innate immune response	123	0.2752	1.03E-27	3.40E-25	Predominantly downregulated	Inflammation / immune response	Il1rap, Mbl2, Adam15, Gbp10, AI182371, Crp, Zbp1, Cd24a
TP Model vs si-Cyp2e1	GO:0006954	inflammatory response	99	0.2870	4.73E-24	1.21E-21	Predominantly downregulated	Inflammation / immune response	Pla2g7, Csrp3, Il1rap, AI182371, Thbs1, Col6a1, Cd14, Vcam1
TP Model vs si-Cyp2e1	GO:0002376	immune system process	131	0.2224	2.78E-20	5.64E-18	Predominantly downregulated	Inflammation / immune response	Ido2, Il1rap, Ctps1, Mbl2, Gbp10, AI182371, Zbp1, Cd24a
TP Model vs si-Cyp2e1	GO:0034097	response to cytokine	30	0.4054	1.65E-12	1.53E-10	Predominantly downregulated	Inflammation / immune response	Col3a1, Ghr, Comt, Lifr, Serpina1d, Kcnj8, Cd38, Slco1b2
TP Model vs si-Cyp2e1	GO:0032760	positive regulation of tumor necrosis factor production	39	0.3171	8.04E-12	6.67E-10	Predominantly downregulated	Inflammation / immune response	Thbs1, Cd14, Il33, Akap12, App, Lrrk2, Oas2, Tgfb1
TP Model vs si-Cyp2e1	GO:0019221	cytokine-mediated signaling pathway	40	0.2740	6.35E-10	3.91E-08	Predominantly downregulated	Inflammation / immune response	Il1rap, Csf2rb2, Ghr, Cebpa, Prlr, Lifr, Lilrb4b, Il4ra
TP Model vs si-Cyp2e1	GO:0006955	immune response	64	0.2006	9.75E-09	4.66E-07	Predominantly downregulated	Inflammation / immune response	Thbs1, Lif, C9, Vtn, Azgp1, Tgfbr3, Cx3cr1, Tlr13
TP Model vs si-Cyp2e1	GO:0032722	positive regulation of chemokine production	13	0.2600	6.63E-04	7.42E-03	Predominantly downregulated	Inflammation / immune response	Il33, App, Hc, Il4ra, Tlr4, Csf1r, F2rl1, Tlr7
TP Model vs si-Cyp2e1	GO:0070098	chemokine-mediated signaling pathway	14	0.2295	1.58E-03	1.42E-02	Predominantly downregulated	Inflammation / immune response	Cx3cr1, Ccr1, Ccr5, Gpr35, Ccl6, Cxcl10, Cxcl5, Ccrl2
TP Model vs si-Cyp2e1	GO:2000379	positive regulation of reactive oxygen species metabolic process	16	0.4103	2.12E-07	7.79E-06	Predominantly downregulated	Oxidative stress / lipid peroxidation	Thbs1, Akr1c14, Nqo2, Tgfbr2, Cyp1b1, Cdkn1a, Plau, Nox4
TP Model vs si-Cyp2e1	GO:0070301	cellular response to hydrogen peroxide	22	0.2895	1.57E-06	4.57E-05	Predominantly downregulated	Oxidative stress / lipid peroxidation	Aqp1, Klf4, Src, Cyp1b1, Ednra, Anxa1, Fabp1, Mdm2
TP Model vs si-Cyp2e1	GO:0006979	response to oxidative stress	31	0.2214	7.50E-06	1.72E-04	Predominantly downregulated	Oxidative stress / lipid peroxidation	Pxdn, Naprt, Gpx3, Cygb, 1600014C10Rik, Abcb11, Comt, Srxn1
TP Model vs si-Cyp2e1	GO:2000377	regulation of reactive oxygen species metabolic process	11	0.3929	2.80E-05	5.24E-04	Predominantly downregulated	Oxidative stress / lipid peroxidation	Bco2, Ier3, Cyp1b1, Lrrk2, Eif6, Prcp, Ngfr, Rnf41
TP Model vs si-Cyp2e1	GO:0034775	glutathione transmembrane transport	5	0.8333	4.44E-05	7.83E-04	Predominantly downregulated	Oxidative stress / lipid peroxidation	Gja1, Abcc4, Abcc1, Slc7a11, Abcc5
TP Model vs si-Cyp2e1	GO:0072593	reactive oxygen species metabolic process	13	0.3023	1.29E-04	1.95E-03	Predominantly downregulated	Oxidative stress / lipid peroxidation	Gls2, Ccn2, Col6a1, Ddit4, Vav1, P2rx7, Nox4, Ucp2
TP Model vs si-Cyp2e1	GO:0034440	lipid oxidation	4	0.5714	2.32E-03	1.87E-02	Mixed	Oxidative stress / lipid peroxidation	Pla2g7, Alox12, Aloxe3, Alox5
TP Model vs si-Cyp2e1	GO:1901299	negative regulation of hydrogen peroxide-mediated programmed cell death	3	0.7500	3.26E-03	2.39E-02	Mixed	Oxidative stress / lipid peroxidation	Hgf, Ddr2, Hk3
TP Model vs	GO:000674	glutathione metabolic process	11	0.2340	4.13E-03	2.88E-02	Mixed	Oxidative stress /	Gstk1, Gstm7, Gstt1, Cth, Gstz1, Gsta3, Slc7a11, Gsr

Comparison	GO ID	GO biological process	Gene count	Rich factor	P value	Adjusted P value / FDR	Direction	Biological category	Representative genes
si-Cyp2e1	9							lipid peroxidation	
TP Model vs si-Cyp2e1	GO:0051897	positive regulation of phosphatidylinositol 3-kinase/protein kinase B signal transduction	64	0.3422	2.14E-20	4.59E-18	Predominantly downregulated	PI3K/AKT-associated signaling	Thbs1, Nrg1, Akr1c14, Src, Cx3cr1, Pdgfra, Trem2, Hgf
TP Model vs si-Cyp2e1	GO:0043491	phosphatidylinositol 3-kinase/protein kinase B signal transduction	26	0.3023	6.92E-08	2.84E-06	Predominantly downregulated	PI3K/AKT-associated signaling	Nrg1, Erbb3, Col6a1, Prex2, Lox, Col6a2, Tmem100, Erbb2
TP Model vs si-Cyp2e1	GO:0051896	regulation of phosphatidylinositol 3-kinase/protein kinase B signal transduction	8	0.2963	2.95E-03	2.29E-02	Predominantly downregulated	PI3K/AKT-associated signaling	Clcf1, Ptpn13, Rnf41, Rapgef3, Rcn3, Cep55, Lime1, Rasgrp1
TP Model vs si-Cyp2e1	GO:0051898	negative regulation of phosphatidylinositol 3-kinase/protein kinase B signal transduction	12	0.2308	3.17E-03	2.39E-02	Predominantly downregulated	PI3K/AKT-associated signaling	Klf4, Trem2, Phlda3, Plekha1, Rapgef1, Serpine2, Mmp3, Pik3ip1
TP Model vs si-Cyp2e1	GO:0043066	negative regulation of apoptotic process	117	0.2212	4.66E-18	7.44E-16	Predominantly downregulated	Regulated cell death / apoptosis	Fstl1, Hspa1b, Rgn, Thbs1, Erbb3, Sgk1, Ada, Aqp1
TP Model vs si-Cyp2e1	GO:0043065	positive regulation of apoptotic process	90	0.2459	3.39E-17	4.89E-15	Predominantly downregulated	Regulated cell death / apoptosis	Nos3, Ccn2, Emilin1, Tbx20, Itga6, Zbp1, Ano6, Frzb
TP Model vs si-Cyp2e1	GO:0006915	apoptotic process	123	0.2050	3.30E-16	4.35E-14	Predominantly downregulated	Regulated cell death / apoptosis	Emp1, 1600014C10Rik, Zbp1, Col6a1, Sgk1, Ada, Tgfbr2, Naip2
TP Model vs si-Cyp2e1	GO:0042981	regulation of apoptotic process	48	0.2308	6.97E-09	3.43E-07	Predominantly downregulated	Regulated cell death / apoptosis	Gls2, Osgin1, Sdf2l1, Acer2, Gja1, Tnfaip8l1, Naip2, Apaf1
TP Model vs si-Cyp2e1	GO:0006629	lipid metabolic process	173	0.2447	1.29E-31	5.43E-29	Mixed	Xenobiotic / lipid metabolism	Sult2a8, Aspg, Pla2g7, Cyp2c23, Hsd3b2, Cyp2c38, Cyp7b1, Hsd17b6
TP Model vs si-Cyp2e1	GO:0008202	steroid metabolic process	49	0.3684	1.84E-17	2.77E-15	Predominantly upregulated	Xenobiotic / lipid metabolism	Sult2a8, Cyp7b1, Hsd17b6, Sc5d, Hsd3b3, Ebp, Ldlr, Insig1
TP Model vs si-Cyp2e1	GO:0008203	cholesterol metabolic process	41	0.3694	6.78E-15	7.84E-13	Predominantly upregulated	Xenobiotic / lipid metabolism	Sult2a8, Ces1e, Cyp7b1, Pon1, Ces1b, Ces1f, Lipc, Ebp
TP Model vs si-Cyp2e1	GO:0006805	xenobiotic metabolic process	36	0.3830	8.12E-14	8.22E-12	Predominantly upregulated	Xenobiotic / lipid metabolism	Sult2a8, Cyp2c54, Cyp2j5, Cyp2c23, Cyp2c38, Cyp2c29, Cyp2c50, Cyp2c67
TP Model vs si-Cyp2e1	GO:0009410	response to xenobiotic stimulus	69	0.2371	1.03E-12	9.74E-11	Predominantly downregulated	Xenobiotic / lipid metabolism	Mmp7, Col1a1, Otc, Thbs1, Hspg2, Col6a1, Maob, Abcb11
TP Model vs si-Cyp2e1	GO:0019373	epoxygenase P450 pathway	19	0.6129	1.90E-12	1.74E-10	Predominantly upregulated	Xenobiotic / lipid metabolism	Cyp2c54, Cyp2j5, Cyp2c23, Cyp2c38, Cyp2c29, Cyp2c50, Cyp2c67, Cyp2c37
TP Model vs si-Cyp2e1	GO:0042908	xenobiotic transport	10	0.4348	2.27E-05	4.38E-04	Mixed	Xenobiotic / lipid metabolism	Abca8a, Gja1, Slc43a3, Slc47a1, Slc22a3, Abca8b, Abcc1, Slc7a5
TP Model vs si-Cyp2e1	GO:0071466	cellular response to xenobiotic stimulus	19	0.2500	7.52E-05	1.25E-03	Predominantly downregulated	Xenobiotic / lipid metabolism	Itgb3, Acer2, Abcb11, Ppp1r9b, Rap1b, Eftud2, Slc10a1, Pde4b

Note: This table summarizes selected enriched GO Biological Process terms from the two RNA-seq comparisons. These terms contextualize broader transcriptomic changes beyond PI3K/AKT signaling, including inflammatory and immune-response programs, oxidative stress- or lipid peroxidation-associated processes, xenobiotic/lipid metabolism, extracellular matrix/cell adhesion remodeling, and regulated cell-death-related programs. These enrichment results should be interpreted as pathway-level associations unless independently validated.
